# Isolation of Heavy Metal-Tolerant and Anti-Phytopathogenic Plant Growth-Promoting Bacteria from Soils

**DOI:** 10.4014/jmb.2407.07013

**Published:** 2024-10-28

**Authors:** Soo Yeon Lee, Kyung-Suk Cho

**Affiliations:** Department of Environmental Science and Engineering, Ewha Womans University, Seoul 03760, Republic of Korea

**Keywords:** Phytoremediation, functional genes, soil bioresource, heavy metal tolerance, plant-growth promoting traits, anti-phytopathogenic activity

## Abstract

In this study, multifunctional soil bacteria, which can promote plant development, resist heavy metals, exhibit anti-phytopathogenic action against plant diseaes, and produce extracellular enzymes, were isolated to improve the effectiveness of phytoremediation techniques. In order to isolate multifunctional soil bacteria, a variety of soil samples with diverse characteristics were used as sources for isolation. To look into the diversity and structural traits of the bacterial communities, we conducted amplicon sequencing of the 16S rRNA gene on five types of soils and predicted functional genes using Tax4Fun2. The isolated bacteria were evaluated for their multifunctional capabilities, including heavy metal tolerance, plant growth promotion, anti-phytopathogenic activity, and extracellular enzyme activity. The genes related to plant growth promotion and anti-phytopathogenic activity were most abundant in forest and paddy soils. *Burkholderia* sp. FZ3 and FZ5 demonstrated excellent heavy metal resistance (≤ 1 mM Cd and ≤ 10 mM Zn), *Pantoea* sp. FC24 exhibited the highest protease activity (24.90 μmol tyrosine·g-DCW^-1^·h^-1^), and *Enterobacter* sp. PC20 showed superior plant growth promotion, especially in siderophore production. The multifunctional bacteria isolated using traditional methods included three strains (FC24, FZ3, and FZ5) from the forest and one strain (PC20) from paddy field soil. These results indicate that, for the isolation of beneficial soil microorganisms, utilizing target gene information obtained from isolation sources and subsequently exploring target microorganisms is a valuable strategy.

## Introduction

In recent years, the rapid advancement in industrial sectors has led to an increased demand for various metals, consequently contributing to the escalating issue of heavy metal contamination [1]. Soil contamination with heavy metals poses a serious risk due to its non-biodegradability and harmful toxicity to both humans and the environment [2]. Although various remediation technologies have been developed to address the removal of highly toxic heavy metals, issues such as high installation costs, maintenance complexity, and the generation of secondary pollutants persist [2]. To overcome the drawbacks of these physical technologies, environmentally friendly approaches such as phytoremediation utilizing plant-plant interactions and rhizoremediation exploiting plant-microbe interactions have been considered as biological restoration methods for purifying heavy metal-contaminated soils [2, 3].

Given that heavy metals hinder the activity of plants and microbes during phytoremediation and rhizoremediation of heavy metal-contaminated soils, recent studies have actively explored the use of bacteria with metal resistance and plant growth-promoting (PGP) abilities to enhance plant growth [4-7]. For example, long-term monitoring (19 months) of *Festuca arundinacea* planted in soil to remove long-lasting residual heavy metals demonstrated the effectiveness of phytoremediation [7]. Moreover, inoculation of the copper- and lead-resistant rhizobacteria *Novosphingobium* sp. CuT1 in soil for the rhizoremediation of heavy metal-contaminated soil improved the growth of *Festuca arundinacea* and increased the availability of heavy metals in the soil [6]. Additionally, the isolation of cadmium-resistant *Enterobacter* sp. FM-1 from cadmium-contaminated soil increased the cadmium content in plants [4], and the rhizosphere bacteria of *Scirpus triqueter* exhibited nickel resistance [5].

During the phytoremediation process, plant pathogens can inhibit plant growth [8]. However, certain soil bacteria play a role in the biological control against plant pathogens, thus protecting plants from disease [8]. Previous studies have shown that *Pseudomonas aeruginosa* and *Pseudomonas fluorescens*, as biological control agents, induce plant systemic resistance (ISR) through the secretion of various plant hormones such as jasmonates and nitrogen oxide, thus protecting plants from pathogens [9]. Particularly, *Pseudomonas* sp. protects plants through its anti-phytopathogenic action, destroying the cell walls of plant pathogens by secreting anti-phytopathogenic substances [10].

Furthermore, efficient decomposition of high molecular weight proteins such as crop residues or animal carcasses present in the soil can reduce the need for nitrogen fertilizers and share nitrogen sources essential for plant growth [11, 12]. Previous studies demonstrated that poultry waste decomposed by *Bacillus* sp. with PGP abilities induced higher growth for carrots, cabbages, and mung beans compared to chemical nitrogen fertilizers [11]. Soil bacteria, especially those involved in promoting plant growth, are suitable for application as biofertilizers, alleviating stress on the soil and plants caused by heavy metal contamination, among other factors [13].

Recently, previous research performed 16S rRNA gene sequencing and predicted functional genes using Tax4Fun2 as an integrated analysis approach. Particularly, it is performed for the isolation and characterization of bacterial resources. In a previous study, genetic information of *Ochrobactrum* sp., possessing plant growth-promoting abilities, was confirmed through genome mining, and its potential for promoting plant growth was evaluated [14]. In another study [15], they identified a cluster of drought-enriched bacteria that significantly correlated with soil compositions. Interestingly, a functional genes analysis by Tax4Fun2 revealed a strong correlation between the same cluster of bacteria and *β*-glucosidase and osmoprotectant proteins, two functions known to be involved in coping with drought stress [15]. However, research on characterizing bacterial resources obtained from those isolation sources identified as having a high potential for obtaining multifunctional microbial resources through the analysis of functional genes using Tax4Fun2 in soil is still lacking.

In this study, soil samples were collected from five regions (abandoned mine, forest, mud flat, paddy field, and wetland) to investigate the functional characteristics of each soil type based on sequencing of the 16S rRNA gene and predicted functional genes using Tax4Fun2. Additionally, multifunctional bacteria were isolated using traditional bacterial isolation methods with soils from the five regions as inoculants. Initially, heavy metal-resistant bacteria were first screened for cadmium and zinc resistance, followed by secondary screening of these strains for PGP, anti-phytopathogenic activity, and extracellular enzyme activity to identify multifunctional bacteria. We aimed to obtain bacterial community structure data for different soil types, isolate multifunctional bacterial resources from soils containing diverse functional genes.

## Materials and Methods

### Soil Sample Collection

To explore useful microbial resources with diverse capabilities, soil samples were collected from five different regions (abandoned mine, forest, mud flat, paddy field, and wetland), and detailed procedures are described in Supplementary materials and methods.

### Isolation of Strains Exhibiting Heavy Metal Resistance, Plant Growth-Promoting Ability, and Anti-Phytopathogenic Activity

**Evaluation of high-concentration heavy metal resistance.** To isolate rhizobacteria with resistance to heavy metals (cadmium and zinc), agar media containing the required heavy metals were prepared [16], and detailed methods are described in the Supplementary materials and methods. The reasons for selecting Cd and Zn among the many types of heavy metals are as follows. Cd is one of the most toxic heavy metals. Zn often exists in an insoluble form and is an essential trace metal, especially for plants [1].

Based on the color and morphology of the colonies grown on each heavy metal agar plate, a total of 192 colonies were selected for further analysis. The 192 selected colonies were inoculated onto Cd-agar plates (0.05, 0.1, 0.5, and 1 mM) and Zn-agar plates (0.5, 1, 5, and 10 mM) to assess their growth. Through this initial heavy metal resistance evaluation, a total of 13 strains were identified. The selected strains were named based on the type of soil they were isolated from (E, F, M, P, and W), the type of heavy metal they were exposed to (C for Cd or Z for Zn), and the order of isolation on each agar plate. For example, the strain isolated from the abandoned mine soil on the 8th spot of the Cd-agar plate was named EC8.

**Evaluation of plant growth-promoting abilities.** The 13 initially selected heavy metal-resistant strains were individually inoculated into 10 ml of LB medium and incubated at 35°C with shaking (120–140 rpm) for 48 h. After centrifugation for 5 min at 5,000 ×*g*, the collected bacterial pellets were resuspended in 10 ml of sterile water and subjected to centrifugation against for bacterial pellet recovery. This washing process was repeated twice. Next, the washed bacterial pellets were resuspended in sterile water to achieve an optical density (OD600) of 1 at 600 nm. The resulting bacterial suspensions were used as inoculants for the evaluation of plant growth-promoting abilities (nitrogen fixation ability, siderophore production, indole-3-acetic acid production, 1-aminocyclopropane-1-carboxylic acid deaminase production and phosphate solubilization ability). The plant growth promotion experiments were conducted with three replicates, and detailed procedures are described in Supplementary materials and methods.

**Evaluation of anti-phytopathogenic activities.** To investigate the anti-phytopathogenic activities of the detailed methods are described in the Supplementary materials and methods.

### Secondary Screening and Taxonomic Identification of Strains Exhibiting Superior Heavy Metal Tolerance, Plant Growth Promotion, and Anti-Phytopathogenic Activity

Four superior strains were identified from the initial 13 based on exceptional metal resistance, plant growth promotion, and anti-phytopathogenic activity [16]. Genomic DNA extraction and partial 16S rRNA gene sequencing were performed, and the determined nucleotide sequences were deposited in GenBank under accession numbers OR775519 (FC24), OR775521 (PC20), OR775531 (FZ3), and OR775579 (FZ5).

A phylogenetic tree, constructed using the BioEdit sequence alignment editor, (version 7.0.5.3), DNA Baser (version 5.15.0.0BT), ClustalX (version 2.1), and MEGA-X (version 10.2.2), elucidated the strains' evolutionary relationships, providing insights into their genetic backgrounds.

### Evaluation of Mineral Solubilization and Extracellular Enzyme Activities of Secondarily Selected Strains

The four strains, identified through the secondary screening, were cultured in 10 ml LB broth at 35°C for 48 h with shaking at 120–140 rpm. After centrifugation and double washing with sterile water, the resulting cell suspensions (OD600 = 1) were used as inocula. All assessments were conducted in triplicate, and detailed procedures are described in Supplementary materials and methods.

### Statistical Analysis

All experimental results were conducted in triplicate, and the mean values along with the standard deviations are presented. The soil microbial community structure was evaluated using a hierarchically clustered heat map (generated using R software, version 4.0.1). For the assessment of anti-phytopathogenic activity, analysis of variance (ANOVA) was performed using R software (version 4.0.1), and post-hoc tests were conducted using the Tukey test at a significance level of *p* < 0.05 after the ANOVA.

## Results

### Bacterial Community Characteristics of Five Soil Samples

The Operational Taxonomic Units (OTUs) and α-diversity metrics analyzed using the Illumina MiSeq method for the five soil samples are presented in Table 1. The Good’s coverage for all samples was above 0.99, indicating a high level of confidence in the analysis results. The number of OTUs in the paddy soil (P sample) was approximately twice as high (4,068) as in soils from the other four regions (2,387~2,989). The number of OTUs in the mud flat (M) soil (2,965) and wetland (W) soil (2,989) was similar, while forest (F) soil showed the lowest number of OTUs (2,387).

In terms of species richness, the Chao1 value for the paddy (P) soil was the highest at 4,370.2, while the abandoned mine (E), mud flat (M), and wetland (W) soils showed similar values ranging from 3,221.2 to 3,309.2. In contrast, the forest (F) soil exhibited a relatively lower Chao1 value of 2,612.4. After calculating the diversity indices, the Shannon and Simpson values for the paddy (P) soil were 10.03 and 0.9978, respectively. The forest (F) soil had the second-highest diversity indices at 9 and 0.9949, followed by wetland (W) soil (8.93, 0.9928), mud flat (M) soil (8.64, 0.9917), and abandoned mine (E) soil (8.23, 0.9878) in descending order (Table 1).

**Fig. 1:**
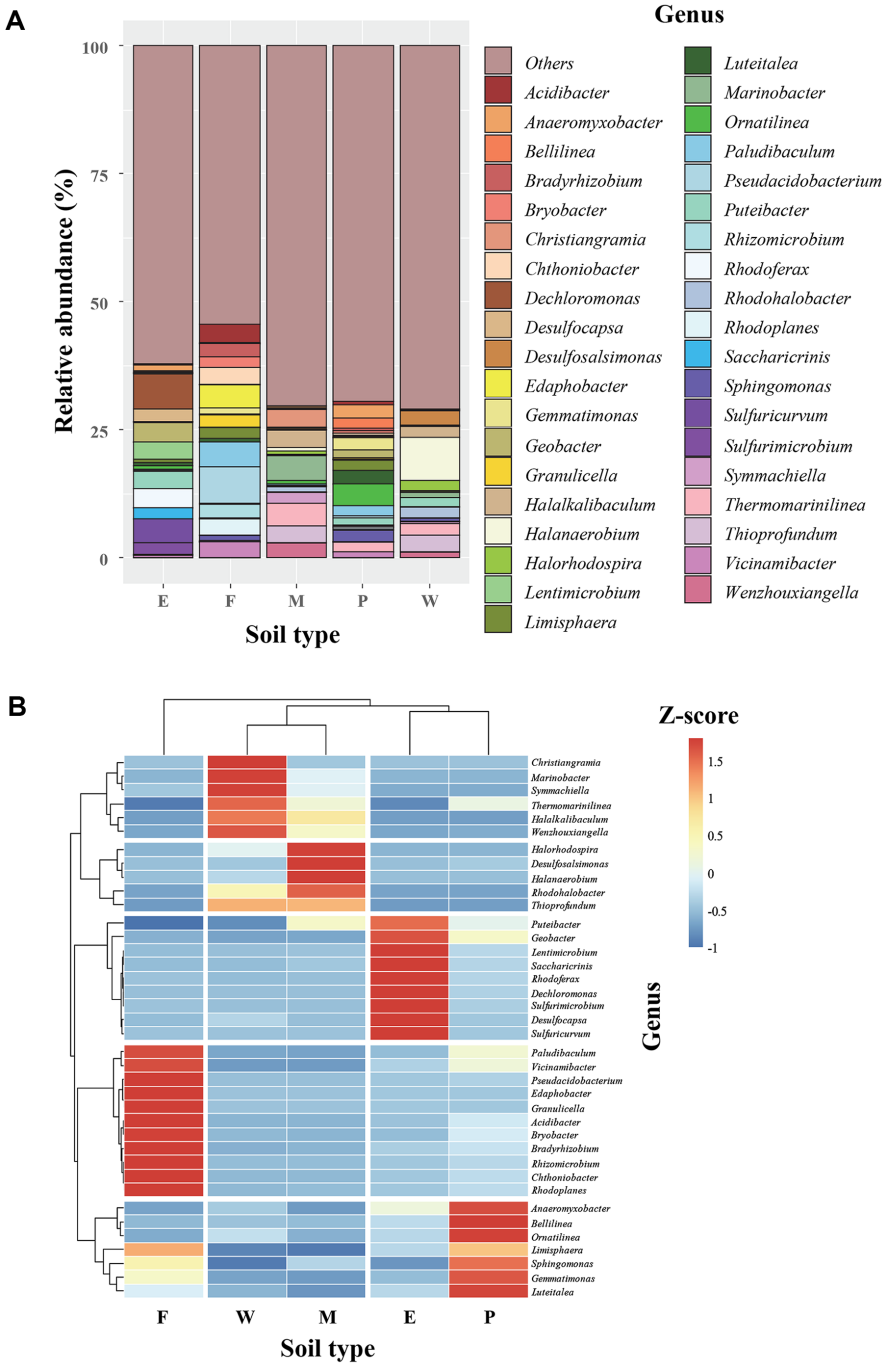
Bacterial community structure dynamics in the E, abandoned mine soil; F, forest soil; M, mudflat soil; P, paddy soil; and W, wetland soil at the genus level (A). Only genera with a relative abundance of >2% are included. Hierarchically clustered heat map of the bacterial distribution at the genus level (**B**). Rows and columns represent bacterial genera and samples, respectively. The relative abundance is represented by color, with the relative abundance increasing from blue to red.

The bacterial community composition for each condition was analyzed at the genus level, considering genera constituting 2% or more of the total genera, as illustrated in Fig. 1A. In the abandoned mine (E) soil sample, three genera, namely *Dechloromonas* (6.87%), *Sulfuricurvum* (4.45%), and *Geobacter* (3.71%), dominated the community. In the forest (F) soil, the predominant genera were *Pseudacidobacterium* (7.10%) and *Paludibaculum* (4.94%). The mud flat (M) soil exhibited richness in *Marinobacter* (4.82%) and *Thermomarinilinea* (4.49%). In the paddy (P) soil, an abundance was observed in *Ornatilinea* (4.24%), *Luteitalea* (2.60%), and *Anaeromyxobacter* (2.58%). The wetland (W) soil displayed high proportions of *Halanaerobium* (8.30%) and *Thioprofundum* (3.28%), with *Thioprofundum* being notably abundant in both the mud flat and wetland soils (3.33%).

The heat map in Fig. 1B illustrates the clustering of microorganisms and the similarity between each sample. The mud flat (M) soil and wetland (W) soil displayed a similar pattern, with a high dominance of *Thioprofundum*, *Halalkalibaculum*, and *Rhodohalobacter* in both soil samples. The abandoned mine (E) and paddy (P) soils also clustered together, primarily influenced by the high abundance of *Anaeromyxobacter* and *Geobacter*. In contrast, the forest (F) soil exhibited a distinctive pattern with a high dominance of *Edaphobacter* and *Granulicella*, distinguishing it from the other soils.

**Fig. 2:**
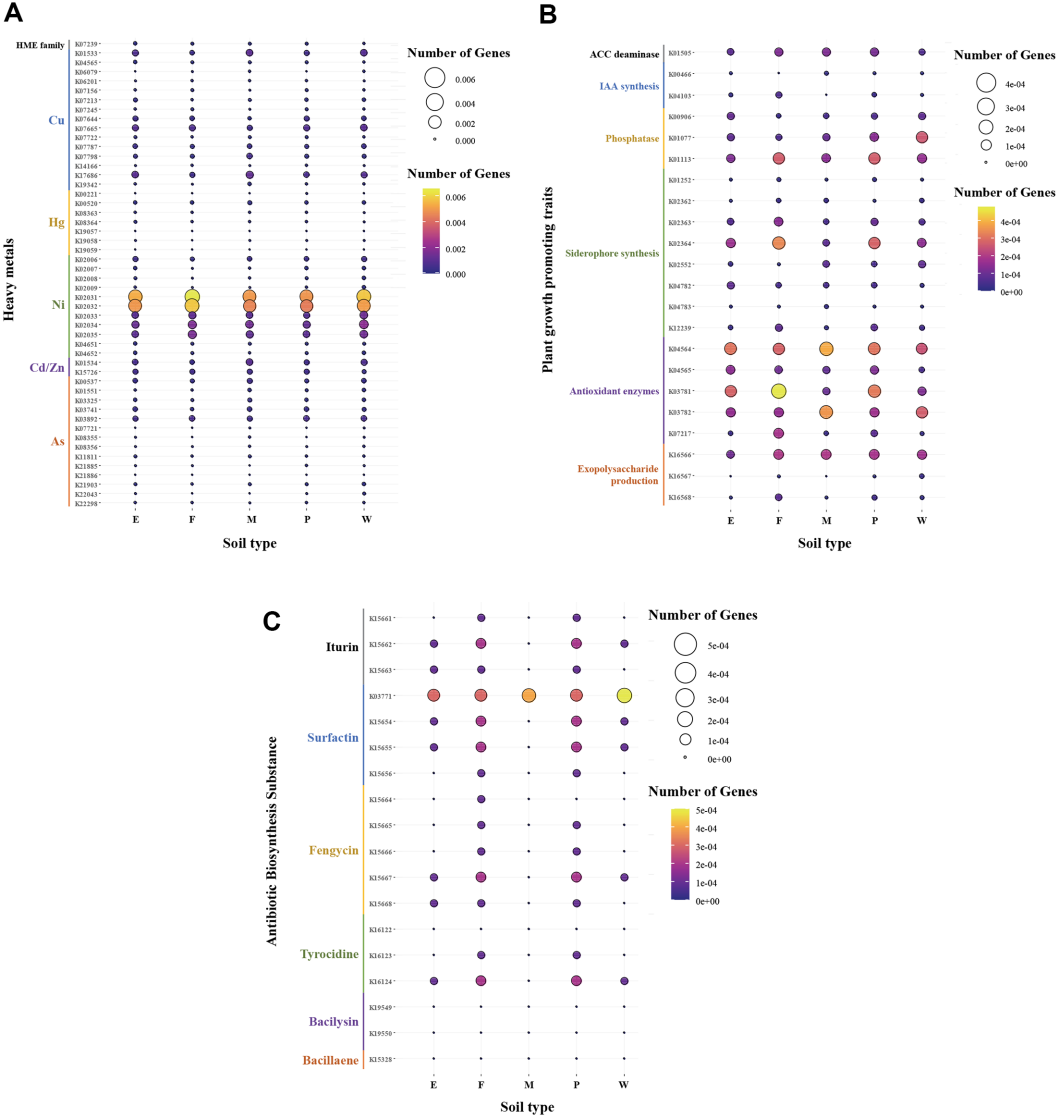
The distribution of genes involved in heavy metals (A) plant growth promotion (B) and antibiotic biosynthesis substances (C) between the Kyoto Encyclopedia of Genes and Genomes (KEGG) ortholog (KO) group and soil types. The size and color of the bubble represent the amount of differentially expressed genes enriched in the five soil samples, respectively.

**Comparison of functional gene distribution within bacterial communities in five soil samples**. We conducted a comparative analysis of the distribution characteristics of functional genes related to heavy metals, plant growth promotion, and anti-phytopathogenic activities based on the bacterial metataxonomic data obtained from soil samples in five regions (abandoned mine, forest, mud flat, paddy field, and wetland) (Table S1-S3 and Fig. 2). A total of 8783 KEGG orthologs (KOs) were identified across the five soil samples. The distribution of genes associated with heavy metal resistance or transport is illustrated in Fig. 2A. The data revealed a nearly uniform relative ratio (2.1–2.5%) of genes related to heavy metals across all soil samples. In most soils, the relative ratio of genes associated with nickel (Ni) transport (K02031 and K02032) was the highest, exceeding 0.4%. Additionally, soil samples from mud flats (M) and wetlands (W) exhibited approximately 1.5–2 times higher relative ratios of genes associated with cadmium and zinc transport (K01534) compared to other soils.

To investigate the potential functions of soil bacteria in promoting plant growth, we selected genes associated with ACC deaminase, IAA synthesis, phosphate solubilization, siderophore synthesis, antioxidant enzymes, and the production of exopolysaccharides (EPS) (Fig. 2B). The relative ratios of genes related to plant growth promotion in the abandoned mine (E) soil sample were relatively lower (17.3%) compared to the gene numbers in other soils (18–24%). Across all soils, the relative ratio of genes involved in IAA synthesis (K00466 and K04103) was around 3%, representing the lowest among genes associated with plant growth promotion. In contrast, genes related to antioxidant enzymes, particularly superoxide dismutase (SOD) activity (K04564), were the most abundant (20%) among the studied genes. In the forest (F) soil sample, genes associated with catalase activity (K03781) were the most abundant (0.05%), while the wetland (W) soil sample exhibited a relatively higher level (0.03%) of genes related to phosphate (K01077).

After investigating the distribution characteristics of potential genes related to anti-phytopathogenic capabilities in soil samples (Fig. 2C), it was observed that all soils harbored the highest abundance of genes (0.03~0.05%) associated with surfactin production (K03771). In contrast, genes related to bacilysin and bacillaene synthesis (K19549, K19550, and K15328) were nearly absent in all soil samples. The relative ratio of genes involved in anti-phytopathogenic capabilities in the mud flat (M) soil sample was the lowest at 9.6%, compared to that of other soil samples. In addition, forest (F) and paddy (P) soils had approximately twice as many genes related to fengycin production (K15667) and tyrocidine synthesis (K16124) compared to those of other soil samples.

### Heavy Metal-Tolerant Bacteria Selection

For comparison with forest and paddy field soil samples selected as multifunctional isolation sources, pure bacteria were also isolated from soil samples from abandoned mines, mudflats, and wetlands, which were the remaining isolation sources. Strains resistant to cadmium (Cd) and zinc (Zn) were selected from soil samples of each of the five regions (abandoned mine, forest, mud flats, paddy field, and wetland). The results showed that abandoned mine soil yielded 23 strains resistant to Cd and 15 strains resistant to Zn; forest soil contained 25 strains resistant to Cd and 23 strains resistant to Zn; mud flat soil had 16 strains resistant to Cd and 13 strains resistant to Zn; paddy soil harbored 24 strains resistant to Cd and 27 strains resistant to Zn; and wetland soil had 21 strains resistant to Cd and five strains resistant to Zn. Out of 192 total isolated strains, 13 strains exhibiting excellent resistance to both Cd and Zn were cross-evaluated and are presented in Table 2. Initial screening identified three strains (EC8, EC17, and EZ11) resistant to Cd and Zn from the abandoned mine soil, four strains (FC24, FZ2, FZ3, and FZ5) from the forest soil, four strains (PC17, PC20, PZ2, and PZ15) from the paddy soil, and two strains (WC11 and WC16) from the wetland soil. Notably, strains FZ2, FZ3, and FZ5 from the forest soil were capable of growth in the highest concentrations of Cd (1 mM) and Zn (10 mM). Strain PZ2 from the paddy soil exhibited growth inhibition in 10 mM Zn but grew at other concentrations. In addition, strains EC17 from the abandoned mine soil and PC20 from the paddy soil grew in 0.1 mM Cd and 5 mM Zn.

### Comparison of PGP Abilities among Primary Selected Heavy Metal-Tolerant Bacteria

The results of evaluating the plant growth-promoting activities of the 13 initially selected heavy metal-tolerant bacterial strains are summarized in Table 2. Bacteria with nitrogen-fixing ability play a role in providing nitrogen to plants in the usable form of nitrate [17]. Except for the EZ11 strain, all 12 strains exhibited nitrogen-fixing ability. Among them, strain PC20 showed the highest nitrogen-fixing activity. Strains EC17, EZ11, and EC8, isolated from the abandoned mine soil, exhibited relatively lower nitrogen-fixing activity compared to strains isolated from other soil types, such as forest, paddy, and wetland soils.

Siderophores produced by bacteria can assist in the absorption of iron by plants, thereby inducing plant growth [18]. Out of 13 total strains, only four strains possessed siderophore-producing capabilities. Among them, PC20 demonstrated the most outstanding siderophore-producing ability, while siderophore activity was also observed in FC24, WC11, and WC16.

Following its nitrogen-fixing and siderophore-producing capabilities, the PC20 strain exhibited the most superior ability in the production of the plant growth hormone IAA [19]. Among strains isolated predominantly from forest soil, FC24, FZ2, FZ3, and FZ5 showed high IAA production capabilities. In contrast, strains EC17, EZ11, and EC8, isolated from abandoned mine soil, demonstrated relatively lower IAA production abilities, exhibiting a trend similar to their nitrogen-fixing abilities.

The ability to degrade the plant stress substance, ACC, a precursor to the plant stress hormone ethylene, was observed in 10 out of 13 bacterial strains, with strains FZ3 and EC8 exhibiting the highest activity [20]. Unlike their IAA production capabilities, strains WC11 and WC16, isolated from wetland soil, showed relatively superior ACC deaminase activity.

Phosphate solubilization ability was observed in all bacterial strains except for six out of 13 strains. Notably, the FZ5 strain exhibited the highest activity with a clear halo exceeding 20 mm. Superior phosphate solubilization abilities were observed in several strains isolated from the forest and paddy soils, including FC24, PC20, PZ15, FZ2, and PZ2.

### Comparison of Anti-Phytopathogenic Activities of Primary Selected Heavy Metal-Tolerant Bacteria

**Fig. 3:**
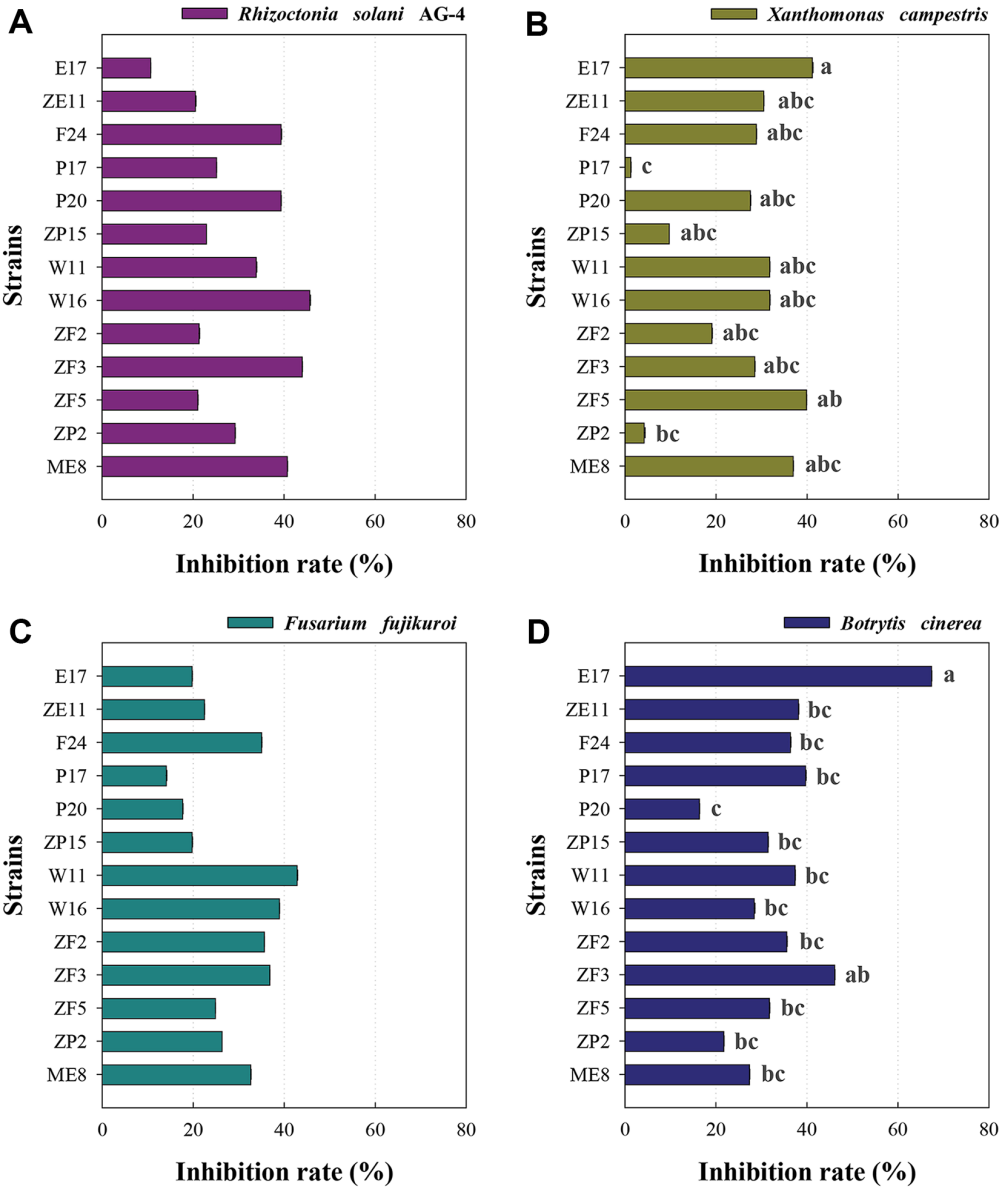
Anti-phytopathogenic activities of the isolates against four different strains of *R. solani* AG-4 (A) *X. campestris* (B) *F. fujikuroi* (C) and *B. cinerea* (D). Anti-phytopathogenic activities were measured based on the size of the zones of inhibition of the pathogen. Zones of inhibition were expressed as percentages. The name of each strain was defined as follows: the first alphabet in the strain name is the type of soil (E, F, M, P, and W), the second alphabet is the type of heavy metal with which it has high tolerance (C: Cd and Z: Zn), and the numbers are the order in which the strain was isolated.

The anti-phytopathogenic effects against four plant pathogens (*R. solani* AG-4, *X. campestris*, *F. fujikuroi*, and *B. cinerea*) of the 13 initially selected strains are presented in Fig. 3. Four plant pathogens have been reported to have adverse effects on crops such as rice and pepper [21-24]. All 13 strains exhibited anti-phytopathogenic activities of 10% or more against *R. solani* AG-4 and *F. fujikuroi*, with no significant differences observed among the strains (Fig. 3A and 3C). Strain E17 showed the highest anti-phytopathogenic activity (41.2%) against *X. campestris* and also demonstrated excellent anti-phytopathogenic activity (67.4%) against *B. cinerea* (Fig. 3B and 3D). While strain ZF5 exhibited relatively high anti-phytopathogenic activity against *X. campestris* (40.3%), strains ZP2 and P17 showed less than 5% anti-phytopathogenic activity (Fig. 3B). Additionally, strain ZF3 displayed a notable anti-phytopathogenic activity of 46.2% against *B. cinerea* (Fig. 3D).

### Identification of Secondary Selected Strains and Their Taxonomic Characteristics

**Fig. 4:**
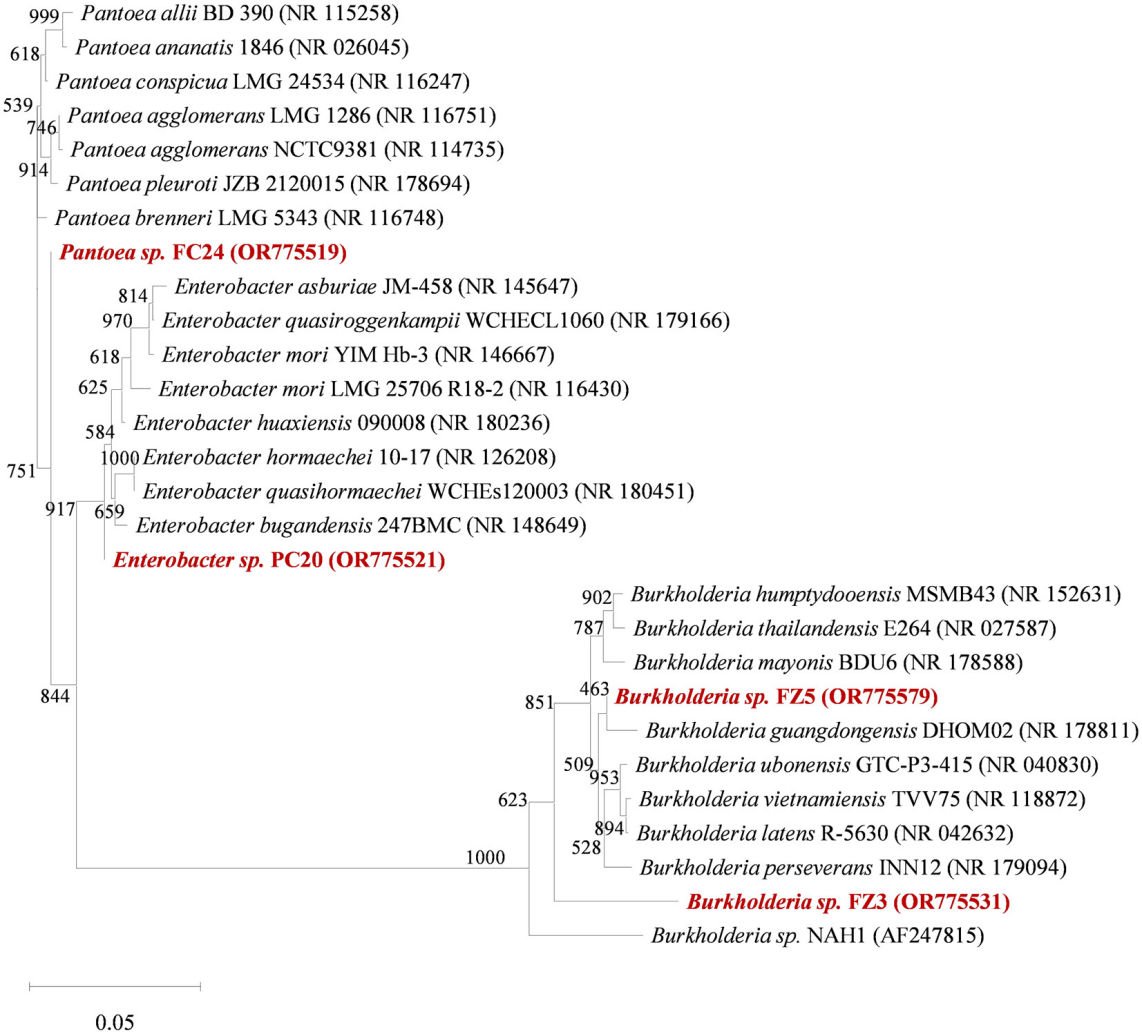
Neighbor-joining phylogenetic tree of strains FC24, PC20, FZ3, and FZ5 estimated from the 16S rRNA sequence. Bootstrap values (percentages of 1,000 replications) are shown at the branch points. The scale bar represents 0.05 substitutions per site. The 16S rRNA gene sequences of bacteria similar to FC24, PC20, FZ3, and FZ5 were used as outgroups.

The results of the identification and phylogenetic characteristics of the four secondary selected strains (FC24, PC20, FZ3, and FZ5), which exhibited superior heavy metal resistance, plant growth-promoting abilities, and anti-phytopathogenic activities among the initially selected strains, are presented in Fig. 4. Strain FC24 (Accession No. OR775519) shared 100% similarity with *Pantoea* sp., known for promoting plant growth in various crops and acting as a biopesticide against plant pathogens [25, 26]. Strain PC20 (Accession No. OR775521) showed 99.8%similarity with *Enterobacter* sp., a species recognized for its high metal resistance and ability to remove and resist metals through cellular mechanisms [27]. Strains FZ3 (Accession No. OR775531) and FZ5 (Accession No. OR775579) exhibited high similarity with *Burkholderia* sp. (93.2% and 93.8%, respectively), and strains within the *Burkholderia* genus have been reported to have anti-phytopathogenic effects against plant pathogens as well as the ability to induce secondary metabolite production for plant growth promotion [28]. Additionally, these strains demonstrated the capability to improve plant growth in cadmium-contaminated soil while accumulating cadmium within cells [29].

### Mineral Solubilization and Extracellular Enzyme Activity of Secondary Selected Strains

Table 3 presents the evaluation results of the mineral solubilization abilities and extracellular enzyme activity of the secondary selected strains in three different types. Regarding calcium carbonate solubilization, no activity was observed in any of the four secondary selected strains. For zinc solubilization, only FC24 and FZ5 exhibited zinc solubilization abilities. Silicon solubilization was most pronounced in PC20, showing the highest activity (>15 mm of clear zone diameter), while FC24 also demonstrated some activity with a clear zone diameter of <15 mm. All four strains exhibited protease activity, with FC24 displaying the highest relative activity at 24.90 μmol tyrosine·g-CDW^-1^·h^-1^. PC20 and FZ3 showed activities of 2.34 and 1.42 μmol tyrosine·g-CDW^-1^·h^-1^, respectively, whereas FZ5 exhibited the lowest protease activity at 0.35 μmol tyrosine·g-CDW^-1^·h^-1^. Consequently, among the secondary selected strains, FC24 demonstrated the ability to solubilize zinc and silicon, along with the most outstanding protease activity.

## Discussion

### Bacterial Community Diversity and Structure

As indicated in Table 1, the species diversity of paddy soil was approximately twice as high as that of the other soil samples. Paddy soil, enriched with nutrients (C, N, and P) from fertilizers and rice straw, which are added to improve crop fertility [30], significantly influenced the diversity of bacterial communities. Consistent with our findings, the bacterial diversity in paddy soil was approximately 1.5 times higher than that in other soils [31].

In our study, the dominant genus in the abandoned mine soil (E) was *Dechloromonas* (Fig. 1). *Dechloromonas* was identified as potential bioindicators for iron, titanium, phosphorus, and organic carbon in the mine soil ecosystem [32]. In contrast, lead and zinc mine soils were dominated by *Blastococcus* and *Nocardioides* [33], while zinc refinery adjacent soil had the predominant genera *Pedomicrobium*, *Bradyrhizobium*, and *Nocardioides* [34]. Heavy metal pollution caused unavoidable changes in the local soil properties and microenvironment [35]. Heavy metals have toxic effects on soil microbial activities and microbial community diversity [35]. There is limited study on the structure and diversity of soil bacterial communities based on contamination levels in abandoned mine soils [36]. Specific exploration is needed to understand the environmental factors that influence bacterial community formation in mine soil ecosystems [36].

The dominant bacteria in major forest soils in Korea were *Actinobacteria*, *Proteobacteria*, and *Planctomycetes* [37, 38], showing differences from the dominant genera in our forest soil (F) (*Pseudacidobacterium* and *Paludibaculum*) (Fig. 1). However, forest soils can harbor a diverse range of bacterial resources due to their varied plant composition and sufficient organic matter content [36]. The dominant bacteria in estuarine mud flat soil (M) were *Marinobacter* and *Thermomarinilinea* (Fig. 1), contrasting with *Vibrio* and *Bacillus* in Incheon estuarine soil in Korea [39]. However, Incheon, the location of soil sampling, is a mud flat habitat where intertidal and subtidal conditions prevail [40]. Therefore, it is expected to harbor a significant presence of bacterial resources primarily adapted to marine environments, such as *Marinobacter* [40]. While *Bradyrhizobium* dominated in wetland soil in Seosan, Korea [41], our study revealed a higher prevalence of *Halanaerobium* and *Thioprofundum* in the same environment (Fig. 1). In the paddy soil (P) of our study, *Ornatilinea* and *Luteitalea* were predominant (Fig. 1), differing from *Streptomyces* and *Nocardioides* found in rice rhizosphere soil in Miryang, South Korea [42]. These results suggest variations in the dominant bacteria depending on the geographical location of soil sampling.

In our study, *Pantoea* sp. FC24, *Burkholderia* sp. FZ3 and FZ5, and *Enterobacter* sp. PC20, isolated as potential microbial resources, did not dominate the soils from which they were isolated (Fig. 4 and Table 3). Moreover, *Pantoea* and *Burkholderia* were present at 0.01% and 0.03%, respectively, in forest soil (F), and *Enterobacter* was present at 0.03% in paddy soil (P) (data not shown).

### Functional Gene

**Heavy metal resistance and transport.** To understand the functional characteristics of soil bacteria in each sample type, we compared the functional gene structure by investigating their distribution related to heavy metal resistance and transport (Fig. 2). Soil bacteria are reported to possess various genes not only associated with resistance and detoxification systems for heavy metals, such as *cad*B, *chr*A, *pbr*A, *Mer*A, and *NiCo*T, but also with transport systems for acquiring these metals [43]. In this study, we investigated the distribution of functional genes related to heavy metal resistance and transport in soil samples from five different regions (Fig. 2A). While we expected a higher relative abundance of functional genes related to heavy metal resistance and transport, such as zinc and arsenic, in the abandoned mine soil (E) sample, a similar trend was observed regardless of soil characteristics. Transport-related genes for nickel, specifically *ddp*D and *ddp*F, were most abundant in all soils (Fig. 2A and Table S1). According to a previous study investigating the abundance of KOs in paddy soil, the abundance of *ddp*D was high [44]. Additionally, *Bacillus* sp. isolated from gold mine soil in Korea was found to harbor genes related to heavy metal transport [45]. In an assessment of heavy metal contamination in major industrial complexes and abandoned mines in Korea, cadmium was identified as the most influential metal, while mercury had the least impact [46]. Notably, it has been reported that heavy metal-containing mine waste, including cadmium, copper, and zinc, accumulates and persists in the soil around abandoned mines in Korea [46]. The detection of heavy metal resistance genes in the metagenomic analysis of soil suggests that various heavy metals are already present in the soil, and some soil microbes residing in these environments possess resistance systems to control heavy metal toxicity [47]. In this study, the predominant residual heavy metals in Korean soils were cadmium, copper, zinc, and nickel, hence, the relative abundance of functional genes related to these metals was relatively higher than that related to arsenic and mercury (Fig. 2A).

**PGP Enzymes.** Abandoned mine soil (E) was expected to have a lower gene abundance related to plant growth promotion due to reduced microbial diversity and impaired microbial community functionality caused by the heavy metal content [48]. Indeed, the abandoned mine (E) soil sample in our study had the lowest number of genes related to plant growth promotion (Fig. 2B and Table S2). Estuarine marsh soil (M) was also inferred to have a lower gene count due to the high salinity affecting microbial community activity in the soil [49]. Wetland (W) soil samples, hosting moisture-tolerant plants, were reported to have relatively diverse functions compared to estuarine marsh (M) soil [49]. Forest (F) soil samples, which exhibited the highest abundance of genes related to plant growth promotion among the five regions (Fig. 2B and Table S2), were influenced by soil bacteria residing in the vicinity, impacting plant growth promotion through IAA metabolism processes [50, 51]. Additionally, paddy (P) soil samples also showed a relatively high number of genes related to plant growth promotion, indicating significantly higher levels of microbial activity and functional diversity in the rhizosphere of rice compared to non-rhizosphere soil [52, 53].

Antioxidant enzymes play a crucial role in reducing plant stress caused by the excess production of ROS due to various environmental factors [54]. They also prevent damage to plant organelles, such as chloroplasts, caused by factors like heavy metals, thus maintaining plant homeostasis [55, 56]. Genes involved in the production of antioxidant enzymes with diverse capabilities were relatively abundant in all soil samples in this study (Fig. 2B). Particularly, the forest soil samples had a high abundance of genes associated with antioxidant enzymes, including catalase activity (CAT) (Fig. 2B), which aligns with previous studies reporting the observation of catalase production in various forest soils [57, 58]. In copper mine soil, the catalase and other antioxidant enzyme activities were low due to heavy metal contamination [59]. Similarly, saline estuarine marsh soil was reported to inhibit antioxidant enzyme activity due to high salinity [60]. In paddy soil samples, catalase activity was increased, possibly due to the addition of straw (an organic carbon source) [61]. In the wetland soil samples, the wet environment was reported to inhibit enzyme activity due to the suppression of plant and microbial survival and interference with the decomposition of organic matter [62].

EPS is a high-molecular-weight polymer composed of sugar moieties and serves as a major component of bacterial cell membranes. It plays a crucial role in alleviating heavy metal stress, enhancing soil aggregation in the rhizosphere, increasing water and nutrient availability for plants, and building plant defense responses [63-65]. Genes involved in EPS production were present in all soil samples at similar levels in this study (Fig. 2B).

Similar to our findings, EPS production has been observed in various soil samples in other studies. In soils from abandoned mines with high concentrations of heavy metals, the soil bacteria communities were found to produce EPS, forming a natural biofilm [66]. Additionally, *Pantoea* sp. isolated from forest and paddy soil samples in other studies was able to produce EPS [65, 67]. Genes related to EPS formation were detected in estuarine mud flat and wetland soil samples as well [68, 69].

**Antibiotic biosynthesis substances.** Among the antibiotic biosynthesis substances, surfactin exhibits broad-spectrum anti-phytopathogenic activity [70, 71]. In our study, genes primarily involved in the production of surfactin and other antibiotic biosynthesis substances (iturin, fengycin, and tyrocidine) were found mainly in forest and paddy soils (Fig. 2C). The *B. cereus* NWUAB01 strain isolated from gold mine soil in South Africa was found to possess genes related to surfactin and fengycin production [45]. Bacteria from wetland sediments and marine environments, which adapt well to extreme conditions such as high salinity, were found to harbor genes related to the production of antibiotic biosynthesis substances [72-74].

### Characteristics of Multifunctional Soil Bacteria for Contaminated Soil Remediation

Initially, during the primary selection of heavy metal-resistant bacteria (192 strains), there was no significant difference in the number of isolated strains among soil samples from the five regions. This observation aligned with the trend in the abundance of metal-associated genes across the five soil sample types, indicating minimal variation between the samples. However, due to the higher abundance of genes related to plant growth promotion and anti-phytopathogenic activity in the forest (F) and paddy (P) soils compared to that of the other three soil types, it was possible to select versatile bacteria primarily from these two soil samples (Fig. 2B and 2C). These results suggest that the strategy of selecting isolates with excellent multifunctionality based on the analysis of functional genes within the five soil types could be effective, especially experimentally screened strains are remediated for soil contaminated with Cd and Zn.

In this study, four outstanding strains were isolated, exhibiting heavy metal resistance, plant growth-promoting (PGP) abilities, and anti-phytopathogenic activity against plant-pathogenic bacteria. *Pantoea* sp. FC24, isolated from forest soil, demonstrated exceptional heavy metal resistance (0.05 mM Cd and 1 mM Zn), PGP traits (nitrogen fixation, siderophore production, IAA synthesis, and phosphate solubilization), mineral solubilization capabilities (zinc and silicon), and high protease activity (Tables 2 and 3). The *Pantoea* species are known for their ability to enhance soil fertility and promote plant growth, making them potential candidates for use as biofertilizers [75]. *Pantoea* sp. DmB 17, isolated from the Himalayan forest soil, exhibited similar characteristics to the FC24 strain, possessing phosphate solubilization and siderophore production abilities [76].

*Pantoea* sp. PP4, isolated from heavy metal-contaminated soil, demonstrated the ability to produce IAA and solubilize inorganic phosphate up to 238 mg/l [77]. This strain also exhibited lead and cadmium resistance and increased the growth of ryegrass (*Lolium multiflorum* Lam) in lead- and cadmium-contaminated soil through a cell adsorption mechanism [77]. *Pantoea agglomerans* isolated from agricultural fields in India exhibited resistance to mercury, copper, arsenic, lead, chromium, and cadmium, along with the ability to accumulate metals inside cells through exopolysaccharide (EPS) production [78]. The *Pantoea* species are well-known for their effectiveness as biological control agents against plant pathogens, producing anti-phytopathogenic compounds such as Herbicolin O and Pantocin A [79-81]. Moreover, *Pantoea* bacteria are reported to enhance plant growth by solubilizing minerals such as silicon, making essential nutrients like phosphorus and potassium available, and using their protease activity [82-84].

*Enterobacter* sp. PC20, isolated from paddy soil, is a strain with heavy metal resistance (0.1 mM Cd and 5 mM Zn), along with PGP abilities such as nitrogen fixation, siderophore production, IAA synthesis, ACC deaminase activity, silicon solubilization capability, and protease activity (Tables 2 and 3). *Enterobacter* sp. FM-1, isolated from heavy metal-contaminated soil, exhibited resistance to cadmium [85]. *E. cloacae* AS10 and *E. aerogenes* K6 isolated from heavy metal-contaminated paddy soil, as well as *E. tabaci* 4M9, isolated from the vicinity of a tin mining area, demonstrated resistance to cadmium, lead, and arsenic [86-88]. *Enterobacter* strains reportedly enhance rice growth and yield by possessing capabilities such as inorganic phosphate solubilization, ACC deaminase activity, nitrogen fixation, and IAA production [86, 87]. Additionally, *Enterobacter* strains have demonstrated anti-phytopathogenic capabilities against various plant pathogens [89, 90]. *Enterobacter ludwigii* GAK2, isolated from paddy soil, demonstrated the ability to solubilize insoluble silicon and promote plant growth in cadmium-contaminated soil [91]. Additionally, *Enterobacter* strains reportedly have the capacity to solubilize zinc and phosphate [92, 93]. These findings highlight the multifunctional and beneficial properties of *Enterobacter* sp. PC20 in promoting plant health and combating soil-borne pathogens.

The FZ3 and FZ5 strains, isolated from forest soil, were identified as *Burkholderia* sp. (Fig. 4). These strains exhibited excellent heavy metal resistance (1 mM Cd and 10 mM Zn) along with plant growth-promoting abilities. Particularly, FZ5 demonstrated outstanding phosphate and zinc solubilization capabilities (Tables 2 and 3). *Burkholderia fungorum* FM-2, isolated from petroleum-contaminated soil, showed resistance to lead, cadmium, and zinc [94]. *Burkholderia contaminans* ZCC, isolated from a copper mine, exhibited cadmium resistance [95], and *Burkholderia cepacia* BAM-12, isolated from mung bean rhizosphere, demonstrated copper, nickel, and lead resistance [96]. *B. cepacia* BAM-12 possesses plant growth-promoting abilities, including IAA production and phosphate solubilization, and inoculation with this strain promoted the growth of rice, corn, and mung bean [96]. The *Burkholderia pyrrocinia* P10 strain, which produces ACC deaminase, enhanced the growth of peanuts upon inoculation [97]. *Burkholderia* sp. JP2-270 harbors the *bysR* gene, which activates antagonistic capabilities. This strain inhibited the growth of *Rhizoctonia solani*, a pathogenic fungus [98]. Additionally, *Burkholderia contaminans* AY001, isolated from weed plants, showed 45% anti-phytopathogenic activity against the plant pathogenic fungus *F. avenaceum* [99]. These findings highlight the diverse functionalities of *Burkholderia* strains in promoting plant growth and protecting against phytopathogens. *Burkholderia* strains isolated from rice rhizosphere were capable of solubilizing insoluble zinc (ZnO, Zn_3_(PO_4_)_2_, and ZnCO_3_) [100]. Additionally, *B. contaminans* AY001, isolated from weed plant rhizospheres, demonstrated zinc and calcium solubilization, along with protease activity [99]. *Burkholderia glumae* was found to harbor the gene *prt*A, which is associated with extracellular enzyme protease activity [101]. These findings emphasize the potential of *Burkholderia* species in environmental bioremediation and plant growth promotion, especially in heavy metal-contaminated soils.

## Conclusion

The analysis of bacterial community structures from soils with diverse characteristics used to isolate multifunctional microorganisms revealed that the bacterial community structures and relative abundance of functional genes were most diverse in forest and paddy soils. Four bacterial strains isolated from forest and paddy field soils were ultimately selected, each possessing heavy metal resistance, plant growth promotion, anti-phytopathogenic capabilities, mineral solubilization ability, and protease activity. These results underscore the importance of actively utilizing gene function information obtained through analysis of 16S rRNA gene sequencing from isolation sources when selecting soil sources for exploring beneficial soil microbial resources. This research suggests that targeting specific soil types rich in multifunctional bacteria and analyzing their gene functions and interactions could be a more effective approach for exploring beneficial soil microbial resources. Adopting a strategy that leverages soils rich in target functional genes as isolation sources can increase the likelihood of securing useful microbial resources while minimizing time and effort. The utilization of these novel microbial resources may serve as innovating biological remediation technologies for soil restoration. Additionally, future studies should investigate the bacterial resources isolated possess functional genes (heavy metal tolerance, plant growth promoting traits and antibacterial properties) and the direct inoculation of the isolated strains into heavy metal-contaminated soils to assess their remediation efficiency and impact on plant growth.

## Supplemental Materials

Supplementary data for this paper are available on-line only at http://jmb.or.kr.



## Figures and Tables

**Table 1:** Operational taxonomic units (OTUs) and alpha-diversity indices for the bacterial soil community.

Sample ^a^	OTUs	Chao1 ^b^	Shannon ^c^	Gini-Simpson ^d^	Good's Coverage ^e^
E	2,807 ± 25.5	3,309.2 ± 19.1	8.23 ± 0.07	0.9878 ± 0.000	0.9913 ± 0.000
F	2,387 ± 28.0	2,612.4 ± 56.6	9 ± 0.04	0.9949 ± 0.000	0.9946 ± 0.000
M	2,965 ± 79.5	3,221.2 ± 68.2	8.64 ± 0.19	0.9917 ± 0.001	0.9961 ± 0.000
P	4,068 ± 126.5	4,370.2 ± 112.3	10.03 ± 0.02	0.9978 ± 0.000	0.9948 ± 0.001
W	2,989 ± 28.5	3,250.9 ± 9.5	8.93 ± 0.03	0.9928 ± 0.000	0.9954 ± 0.000

^a^E, abandoned mine soil; F, forest soil; M, mudflat soil; P, paddy soil; and W, wetland soil

^b^The Chao1 index measures the bacterial population richness.

^c^The Shannon index measures the number and evenness of the species.

^d^The Gini-Simpson index represents the probability that two randomly selected individuals in the habitat will belong to the same species.

^e^Good’s coverage gives a relative measure of how well the sample represents the larger environment. It is calculated as C = 1 − (s − n), where s is the number of unique OTUs and n is the number of individuals in the sample.

**Table 2:** Activities of heavy metal tolerance and plant growth promotion (PGP) by isolated strains.

Strains	Heavy metal tolerance^a^								PGP activity				
	Cd conc. (mM)				Zn conc. (mM)				Nitrogen Fixation (OD_630 nm_)	Siderophore production^b^	IAA production (OD_535 nm_)	ACC deaminase production (OD_600 nm_)	Phosphate solubilization^c^
	0.05	0.1	0.5	1	0.5	1	5	10					
EC8	+	+	-	-	+	+	-	-	0.016 ± 0.006	-	0.175 ± 0.037	0.216 ± 0.002	+
EC17	+	+	-	-	+	+	+	-	0.088 ± 0.005	-	0.691 ± 0.012	0.000 ± 0.005	-
EZ11	-	-	-	-	+	-	-	-	0.000 ± 0.002	-	0.278 ± 0.005	0.000 ± 0.003	-
FC24	+	-	-	-	+	+	-	-	0.122 ± 0.002	+	2.263 ± 0.027	0.000 ± 0.000	++
FZ2	+	+	+	+	+	+	+	+	0.213 ± 0.037	-	1.179 ± 0.016	0.057 ± 0.092	++
FZ3	+	+	+	+	+	+	+	+	0.099 ± 0.079	-	1.371 ± 0.022	0.218 ± 0.010	-
FZ5	+	+	+	+	+	+	+	+	0.123 ± 0.079	-	1.128 ± 0.008	0.110 ± 0.048	+++
PC17	+	+	-	-	+	-	-	-	0.031 ± 0.001	-	0.418 ± 0.007	0.001 ± 0.003	-
PC20	+	+	-	-	+	+	+	-	0.244 ± 0.100	+++	2.990 ± 0.014	0.002 ± 0.002	++
PZ2	+	+	+	+	+	+	+	-	0.085 ± 0.011	-	0.381 ± 0.009	0.043 ± 0.012	++
PZ15	-	-	-	-	+	+	-	-	0.225 ± 0.011	-	1.837 ± 0.007	0.013 ± 0.018	++
WC11	+	+	-	-	+	+	-	-	0.180 ± 0.005	+	0.491 ± 0.001	0.169 ± 0.021	-
WC16	+	-	-	-	+	+	+	-	0.226 ± 0.006	+	0.507 ± 0.006	0.168 ± 0.005	-

^a^-, no growth; +, growth

^b, c^ +++, strong positive (>20 mm of clear zone diameter); ++, positive (10~20 mm); +, weakly positive (<10 mm); -, negative.

IAA, Indole-3-acetic acid; ACC deaminase, 1-aminocyclopropane-1-carboxylic acid deaminase.

**Table 3:** Activities of mineral solubilization and protease from isolated strains.

Strains	Mineral solubilization ^a^			Protease activity (μmol tyrosine·g-DCW^-1^·h^-1^)
	CaCO_3_	Zn_3_(PO_4_)_2_·4H_2_O	Mg_3_H_2_(SiO_3_)_4_	
FC24	-	+	+	24.90 ± 1.45
FZ3	-	-	-	1.42 ± 0.01
FZ5	-	+	-	0.35 ± 0.02
PC20	-	-	++	2.34 ± 0.09

^a^ ++, positive (>15 mm); +, weakly positive (<15 mm); -, negative.

## References

[1] Awa SH, Hadibarata T (2020). Removal of heavy metals in contaminated soil by phytoremediation mechanism: a review. Water Air Soil Pollut..

[2] Yaashikaa PR, Senthil Kumar P, Varjani S, Saravanan A (2020). Rhizoremediation of Cu(II) ions from contaminated soil using plant growth promoting bacteria: an outlook on pyrolysis conditions on plant residues for methylene orange dye biosorption. Bioengineered.

[3] Sharma P, Pandey AK, Udayan A, Kumar S (2021). Role of microbial community and metal-binding proteins in phytoremediation of heavy metals from industrial wastewater. Bioresour. Technol..

[4] Li Y, Liu K, Wang Y, Zhou Z, Chen C, Ye P (2018). Improvement of cadmium phytoremediation by *Centella asiatica* L. after soil inoculation with cadmium-resistant *Enterobacter* sp. FM-1. Chemosphere.

[5] Zhang X, Su C, Liu X, Liu Z, Liang X, Zhang Y (2020). Effect of plant-growth-promoting rhizobacteria on phytoremediation efficiency of *Scirpus triqueter* in pyrene-Ni co-contaminated soils. Chemosphere.

[6] Lee SY, Lee YY, Cho KS (2023). Effect of *Novosphingobium* sp. CuT1 inoculation on the rhizoremediation of heavy metal- and dieselcontaminated soil planted with tall fescue. Environ. Sci. Pollut. Res..

[7] Lee YY, Lee SY, Cho KS (2023). Phytoremediation and bacterial community dynamics of diesel- and heavy metal-contaminated soil: long-term monitoring on a pilot scale. Int. Biodeterior. Biodegradation.

[8] Ortega HE, Torres-Mendoza D, Cubilla-Rios L (2020). Patents on endophytic fungi for agriculture and bio-and phytoremediation applications. Microorganisms.

[9] Gamalero E, Glick BR (2020). The use of plant growth-promoting bacteria to prevent nematode damage to plants. Biology (Basel).

[10] Wang H, Liu R, You MP, Barbetti MJ, Chen Y (2021). Pathogen biocontrol using plant growth-promoting bacteria (PGPR): role of bacterial diversity. Microorganisms.

[11] Jagadeesan Y, Meenakshisundaram S, Raja K, Balaiah A (2023). Sustainable and efficient-recycling approach of chicken feather waste into liquid protein hydrolysate with biostimulant efficacy on plant, soil fertility and soil microbial consortium: a perspective to promote the circular economy. Process Saf. Environ. Prot..

[12] Mekonnen H, Kibret M (2021). The roles of plant growth promoting rhizobacteria in sustainable vegetable production in Ethiopia. Chem. Biol. Technol. Agric..

[13] Borah P, Gogoi N, Asad SA, Rabha AJ, Farooq M (2023). An insight into plant growth-promoting rhizobacteria-mediated mitigation of stresses in plant. J. Plant Growth Regul..

[14] Dilshad R, Mazhar S, Munir S, Jamil N, Batool R (2023). In vitro and in silico study for plant growth promotion potential of indigenous *Ochrobactrum ciceri* and *Bacillus australimaris*. Open Agric..

[15] Si J, Froussart E, Viaene T, Vázquez-Castellanos JF, Hamonts K, Tang L (2021). Interactions between soil compositions and the wheat root microbiome under drought stress: from an in silico to in planta perspective. Comput. Struct. Biotechnol. J..

[16] Lee SY, Lee YY, Cho KS (2021). Characterization of heavy metal tolerant and plant growth-promoting rhizobacteria isolated from soil contaminated with heavy metal and diesel. Microbiol. Biotechnol. Lett..

[17] Kim JY, Kim HS, Lee SM, Park HJ, Lee SH, Jang JS (2020). Plant growth promoting and disease controlling activities of *Pseudomonas geniculata* ANG3, *exiguobacterium acetylicum* ANG40 and *burkholderia stabilis* ANG51 isolated from soil. Microbiol. Biotechnol. Lett..

[18] Choi S, Yoo J-Y, Park S, Park M, Lee O-M, Son H-J (2020). Isolation and characterization of siderophore-producing bacteria with various plant growth-promoting abilities as a potential biocontrol agent. J. Environ. Sci. Int..

[19] Oh KY, Kim JY, Lee SM, Kim HS, Lee KH, Lee SH (2021). Plant growth-promoting activity characteristics of bacillus strains in the rhizosphere. Microbiol. Biotechnol. Lett..

[20] Mou W, Kao YT, Michard E, Simon AA, Li D, Wudick MM (2020). Ethylene-independent signaling by the ethylene precursor ACC in Arabidopsis ovular pollen tube attraction. Nat. Commun..

[21] Holtappels D, Fortuna KJ, Moons L, Broeckaert N, Bäcker LE, Venneman S (2022). The potential of bacteriophages to control *Xanthomonas campestris* pv. *campestris* at different stages of disease development. Microb. Biotechnol..

[22] Kim WG, Shim HS, Lee G Bin, Cho WD (2020). Damping-off of edible amaranth caused by *rhizoctonia solani* AG-4. Korean J. Mycol..

[23] Roca-Couso R, Flores-Félix JD, Rivas R (2021). Mechanisms of action of microbial biocontrol agents against *Botrytis cinerea*. J. Fungi.

[24] Sunani SK, Bashyal BM, Kharayat BS, Prakash G, Krishnan SG, Aggarwal R (2020). Identification of rice seed infection routes of *Fusarium fujikuroi* inciting bakanae disease of rice. J. Plant Pathol..

[25] Khalaf EM, Raizada MN (2020). Draft genome sequences of *Pantoea agglomerans*, *Paenibacillus polymyxa*, and *Pseudomonas* sp. strains, seed biogel-associated endophytes of *Cucumis sativus* L. (Cucumber) and *Cucumis melo* L. (Cantaloupe). Microbiol. Resour. Announc..

[26] Nascimento FX, Hernandez AG, Glick BR, Rossi MJ (2020). The extreme plant-growth-promoting properties of *Pantoea phytobeneficialis* MSR2 revealed by functional and genomic analysis. Environ. Microbiol.

[27] Jiang Z, Jiang L, Zhang L, Su M, Tian D, Wang T (2020). Contrasting the Pb (II) and Cd (II) tolerance of *Enterobacter* sp. via its cellular stress responses. Environ. Microbiol..

[28] Xu Z, Wang M, Du J, Huang T, Liu J, Dong T (2020). Isolation of *Burkholderia* sp. HQB-1, A promising biocontrol bacteria to protect banana against *Fusarium* wilt through phenazine-1-carboxylic acid secretion. Front. Microbiol..

[29] Wang C, Huang Y, Yang X, Xue W, Zhang X, Zhang Y (2020). *Burkholderia* sp. Y4 inhibits cadmium accumulation in rice by increasing essential nutrient uptake and preferentially absorbing cadmium. Chemosphere.

[30] Ding LJ, Su JQ, Sun GX, Wu JS, Wei WX (2018). Increased microbial functional diversity under long-term organic and integrated fertilization in a paddy soil. Appl. Microbiol. Biotechnol..

[31] Wang H, Liu S, Li H, Tao X, Wang H, Qi J (2022). Large-scale homogenization of soil bacterial communities in response to agricultural practices in paddy fields, China. Soil Biol Biochem.

[32] Wang C, Liu S, Wang P, Chen J, Wang X, Yuan Q (2021). How sediment bacterial community shifts along the urban river located in mining city. Environ. Sci. Pollut. Res..

[33] Liu Z, Zhuang J, Zheng K, Luo C (2023). Differential response of the soil nutrients, soil bacterial community structure and metabolic functions to different risk areas in Lead-Zine tailings. Front.Microbiol..

[34] Luo Y, Wu Y, Wang H, Xing R, Zheng Z, Qiu J (2018). Bacterial community structure and diversity responses to the direct revegetation of an artisanal zinc smelting slag after 5 years. Environ. Sci. Pollut. Rse..

[35] Zhao X, Sun Y, Huang J, Wang H, Tang D (2020). Effects of soil heavy metal pollution on microbial activities and community diversity in different land use types in mining areas. Environ. Sci. Pollut. Res..

[36] Wu B, Luo H, Wang X, Liu H, Peng H, Sheng M (2022). Effects of environmental factors on soil bacterial community structure and diversity in different contaminated districts of Southwest China mine tailings. Sci. Total Environ..

[37] Han SI (2016). Phylogenetic characteristics of bacterial populations and isolation of aromatic compounds utilizing bacteria from humus layer of oak forest. Korean J. Microbiol..

[38] Lee B-J, Hyung Eo S (2017). Soil bcterial community in red pine forest of Mt. Janggunbong, Bonghwa-Gun, Gyeongbuk, Korea, using next generation sequencing. J. Korean Forest Soc..

[39] Park J, Shin J, Kim N, Kyoung D, Shim S, Lee JC (2023). Taxonomic characteristics and protease activity exploration of marine heterotrophic bacteria isolated from the coast of Eulwangni in the West Sea, Korea. Korean J. Microbiol..

[40] Park J, Shin J, Kim N, Kyoung D, Shim S, Lee JC (2023). Taxonomic characteristics and protease activity exploration of marine heterotrophic bacteria isolated from the coast of Eulwangni in the West Sea, Korea. Korean J. Microbiol..

[41] Jeong S-Y, Tae, Kim G (2021). Effects of plants on metacommunities and correlation networks of soil microbial groups in an ecologically restored wetland. Microb. Ecol.

[42] Samaddar S, Truu J, Chatterjee P, Truu M, Kim K, Kim S (2019). Long-term silicate fertilization increases the abundance of actinobacterial population in paddy soils. Biol. Fertil Soils.

[43] Xavier JC, Costa PES, Hissa DC, Melo VMM, Falcão RM, Balbino VQ (2019). Evaluation of the microbial diversity and heavy metal resistance genes of a microbial community on contaminated environment. Appl. Geochem..

[44] Huang S, Cao Z, Guan M, Chen M, Lin X (2023). Assessing the ecological impact and microbial restoration of quincloraccontaminated paddy fields through high-throughput sequencing technology. Environ Technol Innov.

[45] Ayangbenro AS, Babalola OO (2020). Genomic analysis of *Bacillus cereus* NWUAB01 and its heavy metal removal from polluted soil. Sci. Rep.

[46] Lee JC, Kang MW, Choi GH, Oh SJ, Kim DJ, Lee SS (2022). Assessment of soil pollutant distribution characteristics and heavy metal pollution in Korea. Korean J. Environ. Agric..

[47] Li S, Zhao B, Jin M, Hu L, Zhong H, He Z (2020). A comprehensive survey on the horizontal and vertical distribution of heavy metals and microorganisms in soils of a Pb/Zn smelter. J. Hazard. Mater..

[48] Yin Y, Wang X, Hu Y, Li F, Cheng H (2023). Soil bacterial community structure in the habitats with different levels of heavy metal pollution at an abandoned polymetallic mine. J. Hazard. Mater..

[49] Zhang X, Liao X, Huang L, Shan Q, Hu A, Yan D (2021). Soil profile rather than reclamation time drives the mudflat soil microbial community in the wheat-maize rotation system of Nantong, China. J. Soil. Sediment..

[50] Larekeng SH, Gusmiaty, Achmad F (2020). Production of IAA hormone in rhizosphere bacterial isolates of community forest stands, IOP Conference Series: Earth and Environmental Science.

[51] Liu J, He X, Sun J, Ma Y (2021). A degeneration gradient of poplar trees contributes to the taxonomic, functional, and resistome diversity of bacterial communities in rhizosphere soils. Int. J. Mol. Sci..

[52] Qu Y, Tang J, Li Z, Zhou Z, Wang J, Wang S (2020). Soil enzyme activity and microbial metabolic function diversity in soda Saline-Alkali rice paddy fields of northeast China. Sustainability (Switzerland).

[53] Flores-Núñez VM, Amora-Lazcano E, Rodríguez-Dorantes A, Cruz-Maya JA, Jan-Roblero J (2018). Comparison of plant growthpromoting rhizobacteria in a pine forest soil and an agricultural soil. Soil Res..

[54] Huchzermeyer B, Menghani E, Khardia P, Shilu A (2022). Metabolic pathway of natural antioxidants, antioxidant enzymes and ROS providence. Antioxidants.

[55] AbdElgawad H, Zinta G, Hamed BA, Selim S, Beemster G, Hozzein WN (2020). Maize roots and shoots show distinct profiles of oxidative stress and antioxidant defense under heavy metal toxicity. Environ. Pollut..

[56] Shah AA, Ahmed S, Ali A, Yasin NA (2020). 2-Hydroxymelatonin mitigates cadmium stress in *cucumis sativus* seedlings: modulation of antioxidant enzymes and polyamines. Chemosphere.

[57] Wan P, He R, Wang P, Cao A (2022). Implementation of different forest management methods in a natural forest: changes in soil microbial biomass and enzyme activities. For Ecol Manage.

[58] Fu Z, Chen Q, Lei P, Xiang W, Ouyang S, Chen L (2021). Soil fungal communities and enzyme activities along local tree species diversity gradient in subtropical evergreen forest. Forests.

[59] Sun H, Zhang J, Wang R, Li Z, Sun S, Qin G (2021). Effects of vegetation restoration on soil enzyme activity in copper and coal mining areas. Environ. Manage..

[60] Feng Y, Xu X, Liu J, Han J, Lu H (2023). Planting *Suaeda salsa* improved the soil properties and bacterial community diversity in a coastal mudflat. Land Degrad. Dev..

[61] Mohamed I, Bassouny MA, Abbas MHH, Ming Z, Cougui C, Fahad S (2021). Rice straw application with different water regimes stimulate enzymes activity and improve aggregates and their organic carbon contents in a paddy soil. Chemosphere.

[62] Zhang Y, Cui D, Yang H, Kasim N (2020). Differences of soil enzyme activities and its influencing factors under different flooding conditions in Ili Valley, Xinjiang. PeerJ..

[63] Dar A, Zahir ZA, Iqbal M, Mehmood A, Javed A, Hussain A (2021). Efficacy of rhizobacterial exopolysaccharides in improving plant growth, physiology, and soil properties. Environ. Monit. Assess..

[64] Zeng W, Li F, Wu C, Yu R, Wu X, Shen L (2020). Role of extracellular polymeric substance (EPS) in toxicity response of soil bacteria *Bacillus* sp. S3 to multiple heavy metals. Bioprocess Biosyst. Eng..

[65] Lv L, Luo J, Ahmed T, Zaki HEM, Tian Y, Shahid MS (2022). Beneficial effect and potential risk of *Pantoea* on rice production. Plants.

[66] Dey S (2022). Indigenous microbial populations of abandoned mining sites and their role in natural attenuation. Arch. Microbiol..

[67] Rocha RT, de Almeida FM, Pappas MCR, Pappas GJ, Martins K (2023). Complete genome sequence of *Pantoea stewartii* RON18713 from Brazil nut tree phyllosphere reveals genes involved in plant growth promotion. Microorganisms.

[68] Mohapatra M, Yadav R, Rajput V, Dharne MS, Rastogi G (2021). Metagenomic analysis reveals genetic insights on biogeochemical cycling, xenobiotic degradation, and stress resistance in mudflat microbiome. J. Environ. Manage..

[69] Cania B, Vestergaard G, Kublik S, Köhne JM, Fischer T, Albert A (2020). Biological soil crusts from different soil substrates harbor distinct bacterial groups with the potential to produce exopolysaccharides and lipopolysaccharides. Microb. Ecol..

[70] Dong H, Gao R, Dong Y, Yao Q, Zhu H (2023). *Bacillus velezensis* RC116 inhibits the pathogens of bacterial wilt and *Fusarium* wilt in tomato with multiple biocontrol traits. Int. J. Mol. Sci..

[71] Kim YS, Lee Y, Cheon W, Park J, Kwon HT, Balaraju K (2021). Characterization of *Bacillus velezensis* AK-0 as a biocontrol agent against apple bitter rot caused by *Colletotrichum gloeosporioides*. Sci. Rep..

[72] Chen Y, Liu SA, Mou H, Ma Y, Li M, Hu X (2017). Characterization of lipopeptide biosurfactants produced by *Bacillus licheniformis* MB01 from marine sediments. Front. Microbiol..

[73] Tian Y, Ji S, Zhang E, Chen Y, Xu G, Chen X (2023). Complete genome analysis of *Bacillus subtilis* TY-1 reveals its biocontrol potential against tobacco bacterial wilt. Mar. Genomics.

[74] Wu S, Liu G, Zhou S, Sha Z, Sun C (2019). Characterization of antifungal lipopeptide biosurfactants produced by marine bacterium *bacillus* sp. CS30. Mar. Drugs.

[75] Shariati VJ, Malboobi MA, Tabrizi Z, Tavakol E, Owilia P, Safari M (2017). Comprehensive genomic analysis of a plant growthpromoting rhizobacterium *Pantoea agglomerans* strain P5. Sci. Rep..

[76] Mittal D, Shukla R, Verma S, Sagar A, Verma KS, Pandey A (2019). Fire in pine grown regions of Himalayas depletes cultivable plant growth promoting beneficial microbes in the soil. Appl. Soil Ecol..

[77] WeiXie L, Yang R, Liu B, Lei N, Peng S, Li J (2022). Effects of Pb-, Cd-resistant bacterium *Pantoea* sp. on growth, heavy metal uptake and bacterial communities in oligotrophic growth substrates of *Lolium multiflorum* Lam. Environ. Sci. Pollut. Res..

[78] Mohite B V., Koli SH, Patil S V (2018). Heavy metal stress and its consequences on Exopolysaccharide (EPS)-producing *Pantoea agglomerans*. Appl. Biochem. Biotechnol..

[79] Matilla MA, Evans TJ, Martín J, Udaondo Z, Lomas-Martínez C, Rico-Jiménez M (2023). Herbicolin A production and its modulation by quorum sensing in a *Pantoea agglomerans* rhizobacterium bioactive against a broad spectrum of plant-pathogenic fungi. Microb. Biotechnol..

[80] Jiang L, Jeong JC, Lee JS, Park JM, Yang JW, Lee MH (2019). Potential of *Pantoea dispersa* as an effective biocontrol agent for black rot in sweet potato. Sci. Rep..

[81] Smits THM, Duffy B, Blom J, Ishimaru CA, Stockwell VO (2019). Pantocin A, a peptide-derived antibiotic involved in biological control by plant-associated *Pantoea* species. Arch. Microbiol..

[82] Sukhwal A, Jain D, Sharma V, Ojha SN, Jat G, K. Upadhyay S (2023). Efficacy evaluation of newly isolated zinc solubilizing bacteria for their potential effect on maize (*Zea mays* L.) under zinc deficient soil conditions. Land Degrad. Dev..

[83] Verma D, Meena RH, Sukhwal A, Jat G, Meena SC, Upadhyay SK (2023). Effect of ZSB with graded levels of zinc fertilizer on yield and zinc uptake under maize cultivation. Proc. Natl. Acad. Sci. India Section B - Biol. Sci..

[84] Lahlali R, Aksissou W, Lyousfi N, Ezrari S, Blenzar A, Tahiri A (2020). Biocontrol activity and putative mechanism of *Bacillus amyloliquefaciens* (SF14 and SP10), *Alcaligenes faecalis* ACBC1, and *Pantoea agglomerans* ACBP1 against brown rot disease of fruit. Microb. Pathog..

[85] Li Y, Mo L, Zhou X, Yao Y, Ma J, Liu K Characterization of plant growth-promoting traits of *Enterobacter* sp. and its ability to promote cadmium/lead accumulation in *Centella asiatica* L. Environ. Sci. Pollut. Res. Int..

[86] Ghosh A, Pramanik K, Bhattacharya S, Mondal S, Ghosh SK, Maiti TK (2022). A potent cadmium bioaccumulating *Enterobacter cloacae* strain displays phytobeneficial property in Cd-exposed rice seedlings. Curr. Res. Microb. Sci..

[87] Mao Y, Tan H, Wang M, Jiang T, Wei H, Xu W (2022). Research progress of soil microorganisms in response to heavy metals in rice. J. Agric. Food Chem..

[88] Abdullahi S, Haris H, Zarkasi KZ, Amir HG (2021). Complete genome sequence of plant growth-promoting and heavy metaltolerant *Enterobacter tabaci* 4M9 (CCB-MBL 5004). J. Basic Microbiol..

[89] Singh RP, Pandey DM, Jha PN, Ma Y (2022). ACC deaminase producing rhizobacterium *Enterobacter cloacae* ZNP-4 enhance abiotic stress tolerance in wheat plant. PLoS One.

[90] Kumar K, Pal G, Verma A, Verma SK (2020). Seed inhabiting bacterial endophytes of finger millet (*Eleusine coracana* L.) promote seedling growth and development, and protect from fungal disease. South Afr. J. Bot..

[91] Adhikari A, Lee KE, Khan MA, Kang SM, Adhikari B, Imran M (2020). Effect of silicate and phosphate solubilizing rhizobacterium *Enterobacter ludwigii* GAK2 on *Oryza sativa* L. under cadmium stress. J. Microbiol. Biotechnol..

[92] Hashemnejad F, Barin M, Khezri M, Ghoosta Y, Hammer EC (2021). Isolation and identification of insoluble zinc-solubilising bacteria and evaluation of their ability to solubilise various zinc minerals. J. Soil Sci. Plant Nutr..

[93] Raturi G, Sharma Y, Mandlik R, Kumawat S, Rana N, Dhar H (2022). Genomic landscape highlights molecular mechanisms involved in silicate solubilization, stress tolerance, and potential growth-promoting activity of bacterium *Enterobacter* sp. LR6. Cells.

[94] Liu X xin, Hu X, Cao Y, Pang W jing, Huang J yu, Guo P (2019). Biodegradation of phenanthrene and heavy metal removal by acid-tolerant *Burkholderia fungorum* FM-2. Front. Microbiol..

[95] You LX, Zhang RR, Dai JX, Lin ZT, Li YP, Herzberg M (2021). Potential of cadmium resistant *Burkholderia contaminans* strain ZCC in promoting growth of soy beans in the presence of cadmium. Ecotoxicol. Environ. Saf..

[96] Popat T, Jha A (2021). *Burkholderia cepacia* BAM-12 isolated from mungbean field in Rajasthan augments plant growth in agricultural field soil of Gujarat, India. J. Microbiol. Biotechnol. Food Sci..

[97] Han L, Zhang H, Xu Y, Li Y, Zhou J (2021). Biological characteristics and salt-tolerant plant growth-promoting effects of an ACC deaminase-producing *Burkholderia pyrrocinia* strain isolated from the tea rhizosphere. Arch. Microbiol..

[98] Song D, Chen G, Liu S, Khaskheli MA, Wu L (2019). Complete genome sequence of *Burkholderia* sp. JP2-270, a rhizosphere isolate of rice with antifungal activity against *Rhizoctonia solani*. Microb. Pathog..

[99] Heo AY, Koo YM, Choi HW (2022). Biological control activity of plant growth promoting rhizobacteria *Burkholderia contaminans* AY001 against tomato *Fusarium* wilt and bacterial speck diseases. Biology (Basel).

[100] Bhakat K, Chakraborty A, Islam E (2021). Characterization of zinc solubilization potential of arsenic tolerant *Burkholderia* spp. isolated from rice rhizospheric soil. World J. Microbiol. Biotechnol..

[101] Lelis T, Peng J, Barphagha I, Chen R, Ham JH (2019). The virulence function and regulation of the metalloprotease gene *prt*A in the plant-pathogenic bacterium *Burkholderia glumae*. Mol. Plant Microbe Interact..

