# Supplementary material for: Isolation of Heavy Metal-Tolerant and Anti-Phytopathogenic Plant Growth-Promoting Bacteria from Soils
Source: J Microbiol Biotechnol. 2024 Oct 28;34(11):2252–65. doi: 10.4014/jmb.2407.07013 (PMC11637846; doi:10.4014/jmb.2407.07013)
Supplement: Supplementary file 1 [file jmb-34-11-2252-supple.pdf]

J. Microbiol. Biotechnol. 2024. 34(11): 2252–2265

https://doi.org/10.4014/jmb.2407.07013

[Supplementary Materials]

Isolation of Heavy Metal-Tolerant and Anti-Phytopathogenic Plant Growth-Promoting Bacteria from Soils

Supplementary Materials and Methods

2.1. Soil sample collection and physicochemical characterization

Soil samples from a closed mine site (E sample) located in Seobuk-gu, Cheonan-si, Chungcheongnam-do (36°91'N, 127°26'E), and the surrounding soils within a 2 km proximity were also collected. Soil samples from the forest soil of Hagyesan in Yangpyeong-gun (F sample) were collected (37°54'N, 127°37'E). Samples from mud flats at the Gaetbeol Experience Center in Oido, Siheung-si, Gyeonggi-do (M sample), and wetland soil (W sample) where sevenleaf mudflower (Chilmyeoncho) plants thrive were collected from the Sohrae Wetland Ecological Park in Oido, Siheung-si (37°41'N, 126°75'E). Soil samples from paddy fields where rice is cultivated in Jatnamugol Road, Jecheon-si, Chungcheongbuk-do (P sample) were also collected (37°17'N, 128°18'E). All soil samples were collected from the surface to a depth of 10 cm, allowed to air-dry for approximately 12–14 hours, and sieved through a 2 mm sieve (DAIHAN®, Seoul, South Korea). Some soil samples were stored at 4°C for physicochemical characterization and microbial community analysis. DNA extraction for microbial community analysis was performed within 24 hours of sample collection. The bacterial community in the soil was analyzed using the Illumina MiSeq sequencing technique [1,2].

The physicochemical properties of the sieved soils, including pH, moisture content, and organic matter content, were analyzed according to the standardized testing methods of South Korea [3] and previous research methods [4,5].

2.1.1. Metagenomic analysis of soil bacterial communities and functional gene analysis by Tax4Fun2

To collect bacterial communities, 0.3 g of sieved soil was stored at -23°C in a sterilized 1.5 mL tube. Genomic DNA was then extracted using the NucleoSpin® Soil Kit (Macherey-Nagel, Düren, Germany) following the manufacturer's guidelines and bead-beating with the FastPrep-24™ (MP Biomedicals, Irvine, CA, USA) system.Next, the extracted DNA, eluted in 50 mL of buffer, was quantified using a SpectraMax QuickDrop spectrophotometer (Molecular Devices, San Jose, CA, USA) and stored at -23°C.

The bacterial communities of the soil samples were analyzed through Illumina MiSeq Sequencing using dual-step PCR with primers 515F (5′- TGC CAG CMG CCG CGG TAA-3′) and 806R (5′-GGA CTA CHV GGG TWT CTA AT-3′), targeting the V4 region of the 16S rRNA gene. The second PCR step was performed according to the manufacturer's instructions

(NexteraXT FC-131-1001, Illumina Inc., CA, USA). For this step, the N7XX (5′-CAAGCAGAAGACGGCATACGAGAT-NNNNNNNN-GTCTCGTGGGCTCGG-3′) and S5XX (5′-AATGATACGGCGACCACCGAGATCTACA-NNNNNNNN-TCGTCGGCAGCGTC-3′) index primer set was used. The underlined, bold, and italicized sequences are adapter sequences, the multiplex identifier tag, and pre-adapters, respectively. Additional information on this process is available in our previous report [2]. Afterward, the purified samples were pooled and analyzed by Macrogen Inc. (Seoul, South Korea) using the Illumina MiSeq Sequencing platform. Sequence reads were then processed by Macrogen Inc. (Seoul, South Korea) using QIIME 1.9software version 1.9 by Macrogen Inc.. Sequences with lengths <200 bp and >400 bp were removed by Fast Length Adjustment of Short reads (FLASH) software version 1.2.11. After then, ambiguous and chimeric sequences were removed and sequences were classified into operational taxonomic units (OTUs) at 97% similarity using CD-HIT-OTU program. The taxonomy for each OTU was assigned based on the NCBI 16S microbial database. The sequencing reads were deposited in the National Centre for Biotechnology Information (NCBI) Sequence Read Archive under accession number SRP480582. Finally, Chao1, Shannon index, and Simpson index were calculated by QIIME software version 1.9.

To conduct functional analysis of bacterial communities in different soils, the genes were compared with the Kyoto Encyclopedia of Genes and Genomes (KEGG) database. The KEGG ortholog (KO) groups used as functional markers in this study are listed in Table S1–S3. The relative abundance of genes related to heavy metal tolerance, plant growth promotion, and antibiotic biosynthesis substances in the soil samples was determined using Tax4Fun2 (https://github.com/bwemheu/Tax4Fun2) [6].

2.2.1. Evaluation of high-concentration heavy metal resistance

Solutions of $CdCl_2 \cdot H_2O$ (Duksan, Ansan-si, Gyeonggi-do, South Korea) and $ZnSO_4 \cdot 7H_2O$ (Duksan) were prepared to a concentration of 1 M each. A sterilized filter (0.45 μm) and a syringe were then used for sterile filtration, and the Cd and Zn heavy metal stock solutions were created. Next, a 1/10 dilution of the 1/10 LB medium (1 g/L tryptone, 0.5 g/L yeast extract, and 1 g/L NaCl) with added agar (15 g/L) was prepared and autoclaved at 120°C for 15 minutes. The sterilized 1/10 LB agar medium was subsequently cooled to around 60°C, and then the Cd or Zn stock solutions were injected to achieve final concentrations of 0.05, 0.1, 0.5, and 1 mM for cadmium, and 0.5, 1, 5, and 10 mM for zinc. The mixture was thoroughly

combined and poured into Petri dishes.

Soil samples (1 g each) from five different regions (abandoned mine, forest, mud flat, paddy field, and wetland) were combined with 9 mL of sterile water in 10 mL tubes. The mixture was stirred at 1,200 rpm for 1 minute, followed by a 15-minute settling period. The supernatant was then collected and stepwise diluted up to 10^{-4} with sterile water, and 200 μL of each diluted soil solution was then spread on heavy metal agar plates. Finally, the plates were cultured at 35°C for 48–72 hours.

2.2.2. Evaluation of plant growth-promoting abilities

Evaluation of nitrogen fixation ability

To assess the nitrogen-fixing capability of the selected strains, a modified version of the nitrogen fixation assay from previous studies was applied [7,8]. Nitrogen-free bromothymol blue (NFB) medium was prepared by adjusting the pH to 7.0 with KOH and NaOH. The NFB medium composition was as follows: 5.0 g malic acid, 0.6 g K_2HPO_4, 0.4 g KH_2PO_4, 0.01 g $MnSO_4$, 0.05 g $MgSO_4$, 0.02 g NaCl, 0.002 g Na_2MoO_4, 2 mL bromothymol blue (0.5% in alcohol), 1000 mL distilled water, and 1.75 g agar. The NFB medium was dispensed in 15 mL tubes (5 mL each) and sterilized under high pressure. Each strain's bacterial suspension (1%, v/v) was then inoculated into the medium, and the tubes were cultured at 20°C for 48 hours. The culture optical density ($OD_{630\ nm}$) was then measured.

Evaluation of siderophore production

The siderophore production of the selected strains was assessed using Chrome Azurol S (CAS) blue agar medium, following the method outlined by the previous study [9–11]. A blue dye solution was prepared by dissolving 60 mg of CAS (Sigma Aldrich Co.) in 50 mL of distilled water, adding 2.7 mg of $FeCl_3 \cdot 6H_2O$ (Sigma Aldrich) to 10 mL of 10 mM HCl, and dissolving 72.9 mg of hexadecyltrimethylammonium bromide (HDTMA, Sigma Aldrich) in 40 mL of distilled water. These three solutions were mixed in the dark to prevent light exposure, and the resulting blue dye solution was sterilized under high pressure (121°C, 15 minutes).

A mixture of 32.24 g of 1,4-Piperazinediethanesulfonic acid (PIPES, Sigma Aldrich) and 15 g of agar in 750 mL of distilled water (pH 6.8) was sterilized under high pressure. After cooling to an appropriate temperature, 30 mL of 10% casamino acid (DifcoTM, Franklin Lakes,

NJ, USA) and 10 mL of 20% glucose were added. To this mixture, 110 mL of distilled water was added to make 900 mL of CAS agar. The previously prepared blue dye solution (100 mL) was then added to achieve a final volume of 1 L. Next, the CAS agar with the blue dye was poured into Petri dishes to create CAS-agar plates. A paper disc with a diameter of 6 mm was then placed in the center of each CAS-agar plate. Afterward, each strain's bacterial suspension (6 μL) was inoculated onto the paper disc. The plates were then incubated at 35°C for 14 days, and the formation of an orange circular zone (orange halo zone) around the paper disc, indicating siderophore production, was observed. Finally, the overall diameter (mm) of the zone was measured.

Evaluation of indole-3-acetic acid (IAA) production

To assess the IAA production capability of the selected strains, each strain's bacterial suspension was inoculated (1%, v/v) into LB medium supplemented with 0.1% L-tryptophan and cultured at 35°C for 24 hours. The culture was mixed with Salkowski reagent (800 μL containing 35% $HClO_4$ and 2 mL of 0.2 M $FeCl_3 \cdot 6H_2O$) and allowed to react in the dark for 30 minutes under anaerobic conditions. The resulting mixture was then centrifuged at 13,200 g for 1 minute, and the supernatant obtained was used to measure the absorbance at 535 nm ($OD_{535\ nm}$) [12].

Evaluation of 1-aminocyclopropane-1-carboxylic acid (ACC) deaminase production

The activity of ACC deaminase, a key indicator of resistance mechanisms to environmental stress, was assessed following the previous study [13,14]. Each strain's bacterial suspension was inoculated (1%, v/v) into DF medium containing ACC instead of $(NH_4)_2SO_4$ as a nitrogen source and cultured at 35°C, 200 rpm for 48 hours. The composition of the DF medium was as follows: 5.8 g Na_2HPO_4, 3 g KH_2PO_4, 0.5 g NaCl, 1 g NH_4Cl, 0.25 mM $CaCl_2$, 1 mM $MgSO_4$, 0.15% glucose, 0.3 mg biotin, and 1,000 mL distilled water. ACC (98%, Sigma-Aldrich, Jerusalem, Israel) was prepared as a 0.5 M stock solution and sterilized through a 0.2 μm filter (Minisart® Syringe filter) before addition to the DF medium to achieve a final concentration of 3 mM. For the control group, an equal volume of each selected strain's bacterial suspension was inoculated into DF medium without added nitrogen, and the culture was incubated at 35°C, 200 rpm for 10 days. The absorbance at 600 nm ($OD_{600\ nm}$) was then measured for both the experimental and control groups. An increase in turbidity indicated the

presence of ACC deaminase activity. The ACC deaminase activity of the selected strains was evaluated by subtracting the absorbance value of the control group from the absorbance value of the experimental group.

Evaluation of phosphate solubilization ability

To assess the phosphate solubilization ability of the selected strains, Pikovskaya's agar medium containing 5 g/L tricalcium phosphate ($Ca_3(PO_4)_2$) was used, following the method by the previous study [8]. The composition of Pikovskaya's medium was as follows: 10.0 g/L glucose, 0.5 g/L $(NH_4)_2SO_4$, 0.2 g/L KCl, 0.1 g/L $MgSO_4$, 0.002 g/L $MnSO_4$, 0.002 g/L $FeSO_4$, 0.5 g/L yeast extract, and 20 g/L agar. After sterilizing the medium, 10 mL was poured into Petri dishes and solidified, and a 6 mm diameter paper disc was placed at the center of each plate. Each selected strain's bacterial suspension (6 μL) was then inoculated onto the paper disc. The plates were then incubated at 35°C for 14 days, and the formation of a clear zone around the paper disc was observed. Finally, the overall diameter of the zone (mm) was measured.

<u>2.2.3. Evaluation of anti-phytopathogenic activities</u>

To investigate the anti-phytopathogenic activities of the selected strains against plant pathogenic bacteria and fungi, one strain of the plant pathogenic bacterium (*Xanthomonas campestris* KACC 10377) and three strains of the plant pathogenic fungi (*Rhizoctonia solani* AG-4 KACC 40141, *Fusarium fujikuroi* KACC 46888, and *Botrytis cinerea* KACC 40573) were obtained from the Korea Agricultural Culture Collection (KACC) and cultured on potato dextrose agar (PD medium; MBcell, Aracaju, Brazil) for 7 days for use in the experiments. These bacteria and fungi were inoculated into 10 mL of PD medium and cultured at 25°C for 24 hours with shaking at 140 rpm. Next, the culture was centrifuged for 5 minutes at 5,000 g to collect the cells, which were resuspended in 10 mL of sterile water and centrifuged again. After repeating this process twice, the washed cells were resuspended in sterile water to an $OD_{600\ nm}$ of 1 to produce a bacterial or fungal suspension for the experiment.

The evaluation of anti-phytopathogenic activities of the selected strains was conducted as follows [15,16]: LB agar and PDA agar were mixed in a 1:1 (v/v) ratio and poured into Petri dishes with a diameter of 90 mm. Two paper discs (6 mm in diameter) were placed 2.5 cm apart, and one disc was inoculated with 6 μL of the bacterial suspension of the selected strain (OD600 = 1), while the other disc was inoculated with 6 μL of the fungal suspension of the

plant pathogenic strain (OD600 = 1). A control group (untreated) was prepared by inoculating only the fungal suspension of the plant pathogenic strain. After incubation at 35°C for 14 days until the fungal mycelium in the control group filled the dish, the distance between the plant pathogenic strain and the selected strain was measured and converted into an inhibition rate using the following equation:

$$Inhibition\, rate\ (\%) = [(R - rt)/rc] * 100$$

R: Distance between the plant pathogenic strain and the isolated strain (mm)
rt: Growth radius in the experimental group (mm)
rc: Growth radius of the plant pathogenic strain in the untreated control group (mm)

2.4. Evaluation of mineral solubilization and extracellular enzyme activities of secondarily selected strains

Evaluation of mineral solubilization ability

For assessing the calcium carbonate solubilization ability of the secondarily selected strains, Deveze-Bruni agar was employed. The composition of the Deveze-Bruni agar medium included 5 g/L D-glucose, 1 g/L yeast extract, 1 g/L peptone, 0.4 g/L K_2HPO_4, 0.01 g/L $MgSO_4$, 5 g/L NaCl, 0.05 g/L $(NH_4)_2SO_4$, 5 g/L $CaCO_3$, and 15 g/L agar (pH 6.8) [17].

To investigate zinc solubilization ability, tris-minimal salt agar was utilized, consisting of 10 g/L D-glucose, 6.06 g/L Tris-HCl, 4.68 g/L NaCl, 1.49 g/L KCl, 1.07 g/L NH_4Cl, 0.43 g/L Na_2SO_4, 0.2 g/L $MgCl_2{\cdot}2H_2O$, 0.03 g/L $CaCl_2{\cdot}2H_2O$, 1.998 g/L $Zn_3(PO_4)_2{\cdot}4H_2O$, and 15 g/L agar (pH 7.0) [17].

For the silicon solubilization ability assessment, silicate agar was employed, containing 10 g/L D-glucose, 2.5 g/L magnesium trisilicate, and 15 g/L agar (pH 6.8) [18].

A 6 mm paper disc was centrally placed on each of the three agar plates, and 6 μL of the secondarily selected strains' cell suspensions were inoculated. Following incubation at 35°C for 7 days, the formation of a clear zone around the bacterial colonies was observed, and the overall diameter of the zone was measured.

Evaluation of extracellular enzyme activity

The protease activity of the secondarily selected strains' cells was evaluated as follows [19–21]. In a 15 mL test tube, 1 mL of the bacterial cell suspension and 1 mL of 0.2 M Tris

buffer (pH 8.0) were mixed (1:1, v/v). Subsequently, 1 mL of 2% sodium caseinate was added, and the mixture was thoroughly blended. The test tube was then sealed with parafilm to prevent evaporation and incubated in a water bath at 50°C for 2 hours. To stop the reaction, 1 mL of 10% trichloroacetic acid was added, and the mixture was incubated at 35°C for 10 minutes to coagulate the proteins. The supernatant (0.5 mL) obtained by centrifugation at 13,000 rpm for 1 minute was then transferred to a new 15 mL test tube. Then, 0.75 mL of 1.4 M Na_2CO_3 and 0.25 mL of 33% Folin-Ciocalteu's phenol reagent (Sigma-Aldrich, St. Louis, MO, USA) were added to the test tube. After the mixture remained at room temperature for 10 minutes, the absorbance was measured at 650 nm. Protease activity was calculated from a standard curve prepared by quantifying tyrosine (Sigma-Aldrich) [22,23]. The protease activity of the selected strains was compared based on the rate of tyrosine production per unit dry cell weight (μmol tyrosine·g-DCW^{-1}·h^{-1}). The cell dry weight was determined by centrifuging (5,000 g, 5 min) 100 mL of the culture, drying the settled cell mass for 4 hours (105°C), and measuring the weight difference after sufficient cooling at room temperature.

Table S1. Full list of Kyoto Encyclopedia of Genes and Genomes (KEGG) Orthologs (KOs) involved in potential pathways for heavy metal resistance in the five soil samples (https://www.genome.jp/kegg/genes.html).

Potential pathways heavy metal resistance	KO number	Full gene name
HME family	K07239	TC.HME; heavy-metal exporter, HME family
Cu	K01533	*cop* B; Cu^{2+}-exporting ATPase
	K04565	SOD1; superoxide dismutase, Cu-Zn family
	K06079	*cut* F, *nlp* E; copper homeostasis protein (lipoprotein)
	K06201	*cut* C; copper homeostasis protein
	K07156	*cop* C, *pco* C; copper resistance protein C
	K07213	ATOX1, ATX1, *cop* Z, *gol* B; copper chaperone
	K07245	*pco* D; copper resistance protein D
	K07644	*cus* S, *cop* S, *sil* S; two-component system, OmpR family, heavy metal sensor histidine kinase CusS
	K07665	*cus* R, *cop* R, *sil* R; two-component system, OmpR family, copper resistance phosphate regulon response regulator CusR
	K07722	*nik* R; *cop* G family transcriptional regulator, nickel-responsive regulator
	K07787	*cus* A, *sil* A; Cu(I)/Ag(I) efflux system membrane protein CusA/SilA
	K07798	*cus* B, *sil* B; membrane fusion protein, Cu(I)/Ag(I) efflux system
	K14166	*ycn* J; copper transport protein
	K17686	*cop* A, *ctp* A, ATP7; Cu^{+}-exporting ATPase
	K19342	*nos* L; copper chaperone NosL
Hg	K00221	alkylmercury lyase
	K00520	*mer* A; mercuric reductase
	K08363	*mer* T; mercuric ion transport protein
	K08364	*mer* P; periplasmic mercuric ion binding protein
	K19057	*mer* D; MerR family transcriptional regulator, mercuric resistance operon regulatory protein
	K19058	*mer* C; mercuric ion transport protein
	K19059	*mer* E; mercuric ion transport protein
Ni	K02006	*cbi* O; cobalt/nickel transport system ATP-binding protein
	K02007	*cbi* M; cobalt/nickel transport system permease protein
	K02008	*cbi* Q; cobalt/nickel transport system permease protein
	K02009	*cbi* N; cobalt/nickel transport protein
	K02031	*ddp* D; peptide/nickel transport system ATP-binding protein
	K02032	*ddp* F; peptide/nickel transport system ATP-binding protein
	K02033	*ddp* B; peptide/nickel transport system permease protein
	K02034	*ddp* C; peptide/nickel transport system permease protein
	K02035	*ddp* A, *ygi* S; peptide/nickel transport system substrate-binding protein
	K04651	*hyp* A, *hyb* F; hydrogenase nickel incorporation protein HypA/HybF
	K04652	*hyp* B; hydrogenase nickel incorporation protein HypB
Cd/Zn	K01534	*znt* A; Cd^{2+}/Zn^{2+}-exporting ATPase
	K15726	*czc* A, *cus* A, *cnr* A; cobalt-zinc-cadmium resistance protein CzcA
As	K00537	*ars* C; arsenate reductase
	K01551	*ars* A, ASNA1, GET3; arsenite/tail-anchored protein-transporting ATPase
	K03325	ACR3, *ars* B; arsenite transporter
	K03741	*ars* C; arsenate reductase
	K03892	*ars* R; ArsR family transcriptional regulator, arsenate/arsenite/antimonite-responsive transcriptional repressor
	K07721	ArsR family transcriptional regulator
	K08355	*aox* A; arsenite oxidase small subunit
	K08356	*aox* B; arsenite oxidase large subunit
	K11811	*ars* H; arsenical resistance protein ArsH
	K21885	*cmt* R; ArsR family transcriptional regulator, cadmium/lead-responsive transcriptional repressor
	K21886	*nmt* R; ArsR family transcriptional regulator, nickel/cobalt-responsive transcriptional repressor
	K21903	*cad* C, smtB; ArsR family transcriptional regulator, lead/cadmium/zinc/bismuth-responsive transcriptional repressor
	K22043	*czr* A; ArsR family transcriptional regulator, zinc-responsive transcriptional repressor
	K22298	*smt* B; ArsR family transcriptional regulator, zinc-responsive transcriptional repressor

Table S2. Full list of Kyoto Encyclopedia of Genes and Genomes (KEGG) Orthologs (KOs) involved in potential pathways for plant growth promotion in the five soil samples (https://www.genome.jp/kegg/genes.html).

Potential pathways for plant growth promotion	KO number	Full gene name
**ACC deaminase**	K01505	*acd*S; 1-aminocyclopropane-1-carboxylate deaminase
**IAA synthesis**	K00466	*iaa*M; tryptophan 2-monooxygenase
	K04103	*ipd*C; indolepyruvate decarboxylase
**Phosphatase**	K00906	*ace*K; isocitrate dehydrogenase kinase/phosphatase
	K01077	*pho*A, *pho*B; alkaline phosphatase
	K01113	*pho*D; alkaline phosphatase D
**Siderophore synthesis**	K01252	*ent*B, *dhb*B, *vib*B, *mxc*F; bifunctional isochorismate lyase/aryl carrier protein
	K02362	*ent*D; enterobactin synthetase component D
	K02363	*ent*E, *dhb*E, *vib*E, *mxc*E; 2,3-dihydroxybenzoate-AMP ligase
	K02364	*ent*F; enterobactin synthetase component F
	K02552	*men*F; menaquinone-specific isochorismate synthase
	K04782	*pch*B; isochorismate pyruvate lyase
	K04783	*irp*5, *ybt*E; yersiniabactin salicyl-AMP ligase
	K12239	*pch*E; dihydroaeruginoic acid synthetase
**Antioxidant enzymes**	K04564	superoxide dismutase, Fe-Mn family
	K04565	superoxide dismutase, Cu-Zn family
	K03781	*kat*E, CAT, *cat*B, *srp*A; catalase
	K03782	*kat*G; catalase-peroxidase
	K07217	Mn-containing catalase
**Exopolysaccharide production**	K16566	*exo*Y; exopolysaccharide production protein ExoY
	K16567	*exo*Q; exopolysaccharide production protein ExoQ
	K16568	*exo*Z; exopolysaccharide production protein ExoZ

IAA, Indole-3-acetic acid; ACC deaminase, 1-aminocyclopropane-1-carboxylic acid deaminase.

Table S3. Full list of Kyoto Encyclopedia of Genes and Genomes (KEGG) Orthologs (KOs) involved in potential pathways for antibiotic biosynthesis substances in the five soil samples (https://www.genome.jp/kegg/genes.html).

Potential pathways antibiotic biosynthesis substance	KO number	Full gene name
**Iturin**	K15661	*itu*A, *myc*A, *bmy*A; iturin family lipopeptide synthetase A
	K15662	*itu*B, *myc*B, *bmy*B; iturin family lipopeptide synthetase B
	K15663	*itu*C, *myc*C, *bmy*C; iturin family lipopeptide synthetase C
**Surfactin**	K03771	*sur*A; peptidyl-prolyl cis-trans isomerase SurA
	K15654	*srf*AA, *lic*A, *lch*AA; surfactin family lipopeptide synthetase A
	K15655	*srf*AB, *lic*B, *lch*AB; surfactin family lipopeptide synthetase B
	K15656	*srf*AC, *lic*C, *lch*AC; surfactin family lipopeptide synthetase C
**Fengycin**	K15664	*pps*A, *fen*C; fengycin family lipopeptide synthetase A
	K15665	*pps*B, *fen*D; fengycin family lipopeptide synthetase B
	K15666	*pps*C, *fen*E; fengycin family lipopeptide synthetase C
	K15667	*pps*D, *fen*A; fengycin family lipopeptide synthetase D
	K15668	*pps*E, *fen*B; fengycin family lipopeptide synthetase E
**Tyrocidine**	K16122	*tyc*A; tyrocidine synthetase I
	K16123	*tyc*B; tyrocidine synthetase II
	K16124	*tyc*C; tyrocidine synthetase III
**Bacilysin**	K19549	*bac*F; bacilysin biosynthesis transaminase BacF
	K19550	*bac*G; bacilysin biosynthesis oxidoreductase BacG
**Bacillaene**	K15328	*pks*D, *bae*D; bacillaene synthase trans-acting acyltransferase

Supplementary Results and Discussion

At the genus level, the taxonomic composition of the top 10 genera and their associations based on Spearman correlation coefficients at the 0.4 level ($p < 0.01$) relative to the abundance of OTUs were presented for each soil sample (Fig. S1). In the abandoned mine (E) soil, *Sulfuricurvum* dominated, showing significant positive associations (21.1%) with *Geobacter*, *Puteibacter*, and *Saccharicrinis* (Fig. S1a). *Pseudacidobacterium* was the dominant genus in the forest (F) soil, exhibiting significant positive associations (26.7% and 17.8%) with *Paludibaculum* and *Edaphobacter*, respectively (Fig. S1b). In the paddy (P) soil, *Ornatilinea*, the dominant genus, showed a significant positive association (15.4%) with *Bellilinea* and *Anaeromyxobacter* (Fig. S1d). Mud flat (M) and wetland (W) soils harbored *Thioprofundum*, *Halanaerobium*, and *Marinobacter* concurrently, exhibiting nearly identical taxonomic compositions (Fig. S1c and e). In the mud flat (M) soil, *Wenzhouxiangella* was the dominant genus, with significant positive associations (17.6% and 11.8%) with *Thioprofundum* and *Marinobacter*, respectively (Fig. S1c). Additionally, the wetland (W) soil was dominated by *Thermomarinilinea*, showing the highest positive association (17.3%) with *Halalkalibaculum* (Fig. S1e).

Consistent with a previous study [24], our results indicated the coexistence of *Marinobacter* and *Wenzhouxiangella* in the wetland sediment, particularly with a correlation involving the denitrification gene (*nir*S). Our study also revealed similar taxonomic compositions in the coexistence of these two genera in the marsh soil (Fig. S1c). In another study [25], *Ornatilinea* and *Anaeromyxobacter* coexisted in arsenic-contaminated rice rhizosphere soil. In a long-term observation with fertilizer application, *Anaeromyxobacter* increased in relative abundance over time, whereas *Ornatilinea* decreased, contrary to our findings, suggesting a negative correlation over time (Fig. S1d).

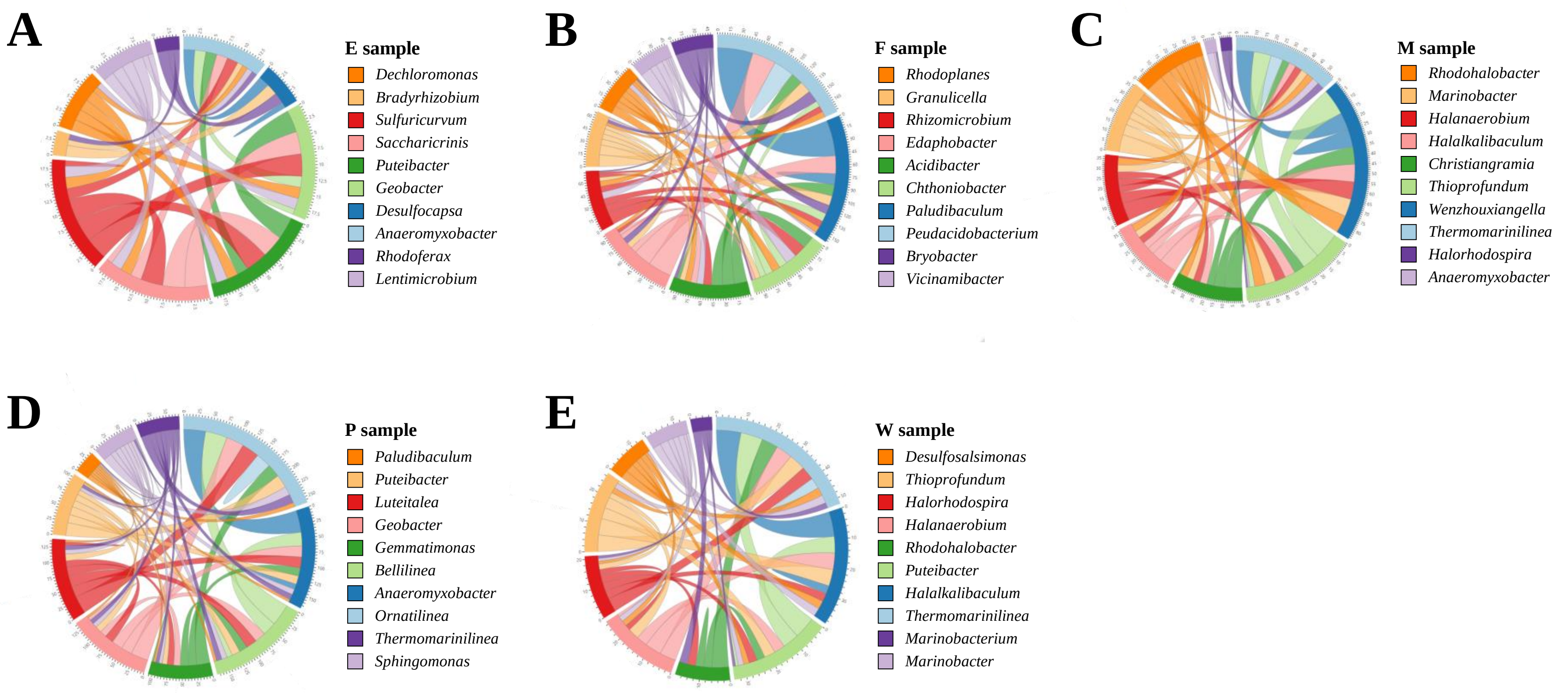

Fig. S1 Chord-diagram visualizing the taxonomy-positive relationships of Spearman correlation values (cutoff = 0.4, *p*-value≤0.01) between the top 10 genera in the abandoned mine (E) soil (a), forest (F) soil (b), mud flat (M) soil (c), paddy (P) soil (d) and wetland (W) soil (e).

References

1. Lee YY, Lee SY, Cho KS. 2023. Phytoremediation and bacterial community dynamics of diesel- and heavy metal-contaminated soil: Long-term monitoring on a pilot scale. *Int Biodeterior Biodegradation* **183**: 105642.

2. Lee SY, Lee YY, Cho KS. 2023. Inoculation effect of heavy metal tolerant and plant growth promoting rhizobacteria for rhizoremediation. *International Journal of Environmental Science and Technology* **21**: 1419-1434.

3. Korean Ministry of Environment. 2018. Korean standard soil analysis method. http://me.go.kr/home/web/law/read.do?pagerOffset=0&maxPageItems=10&maxIndexPages=10&searchKey=lawTitle&searchValue=%ED%86%A0%EC%96%91&menuId=71&orgCd=&condition.typeCode=admrul&typeCode=admrul &lawSeq=586. (Accessed 16 January 2023).

4. Kim S-R, Lee S-C, Jung J-W, Park S-H, Hong S-H, Lee B-O, *et al.* 2023. Characteristics of compost produced from Hanwoo (Korean native cattle) farm. *J. Anim. Environ. Sci* **25**: 22–28.

5. Park SH, Kim JW, Jeon SW, Park HM, Jung MC. 2022. Case Study on Evaluated Soil Health Properties Before and After Land Farming and Thermal Desorption for TPH-contaminated Soil. *Journal of the Korean Society of Mineral and Energy Resources Engineers* **59**: 333–345.

6. Wemheuer F, Taylor JA, Daniel R, Johnston E, Meinicke P, Thomas T, *et al.* 2020. Tax4Fun2: Prediction of habitat-specific functional profiles and functional redundancy based on 16S rRNA gene sequences. *Environmental Microbiomes* **15**: 1-12.

7. de Oliveira DM, de Lima ALA, Diniz NB, Santos CE de R e. S, da Silva SLF, Simões A do N. 2018. Inoculation of plant-growth-promoting rhizobacteria in *Myracrodruon urundeuva* Allemão supports in tolerance to drought stress. *J Plant Interact* **13**: 91–99.

8. Kim Y-K, Hong S-J, Shim C-K, Kim M-J, Choi E-J, Lee M-H, *et al.* 2012. Functional Analysis of *Bacillus subtilis* Isolates and Biological Control of Red Pepper Powdery Mildew Using *Bacillus subtilis* R2-1. *Research in Plant Disease* **18**: 201–209.

9. Kannahi M, Senbagam N. 2014. Studies on siderophore production by microbial isolates obtained from rhizosphere soil and its antibacterial activity. *Journal of Chemical and Pharmaceutical Research* **6**: 1142–1145.

10. Louden BC, Haarmann D, Lynne AM. 2011. Use of Blue Agar CAS Assay for Siderophore Detection. *J Microbiol Biol Educ* **12**: 51–53.

11. Srimathi K, Suji HA. 2018. Siderophores Detection by using Blue Agar CAS Assay Methods. *Biological Sciences* **5**: 180–185.

12. Lee YJ, Ganbat D, Jeong GE, Shin KS, Lee SJ. 2022. A Study on the Isolation and Characterization of Aerobic Halophilic Microorganisms Isolated from the Soil Around the Port on Jeju Island. *Food Engineering Progress* **26**: 140–146.

13. Dell'Amico E, Cavalca L, Andreoni V. 2005. Analysis of rhizobacterial communities in perennial *Graminaceae* from polluted water meadow soil, and screening of metal-resistant, potentially plant growth-promoting bacteria. *FEMS Microbiol Ecol* **52**: 153–162.

14. Grobelak A, Kokot P, Światek J, Jaskulak M, Rorat A. 2018. Bacterial ACC deaminase activity in promoting plant growth on areas contaminated with heavy metals. *Journal of*

Ecological Engineering **19**: 150–157.

15. Antonio G, Lirio C, Coronado AS, Labana R V, Dungca JZ, Cabrera E-RC, *et al.* 2022. Antimicrobial activity of the Rhizospheric *Bacillus* species isolated from Potato (*Solanum tuberosum*) Organic Farm Soils in the Philippines. *European Online Journal of Natural and Social Sciences* **11**: 1–174.

16. Sawant SS, Song J, Seo HJ. 2022. Characterization of *Bacillus velezensis* RDA1 as a Biological Control Agent against White Root Rot Disease Caused by *Rosellinia necatrix*. *Plants* **11**: 2486.

17. Oh KY, Kim JY, Lee SM, Kim HS, Lee KH, Lee SH, *et al.* 2021. Plant growth-promoting activity characteristics of *Bacillus* strains in the rhizosphere. *Microbiology and Biotechnology Letters* **49**: 403–413.

18. Kim HS, Oh KY, Lee SM, Kim JY, Lee KH, Lee SH, *et al.* 2021. Comparison of Antifungal Activity, Plant Growth Promoting Activity, and Mineral-Solubilizing Ability of *Bacillus* sp. Isolated from Rhizosphere Soil and Root. *Microbiology and Biotechnology Letters* **49**: 576–586.

19. Ahn Y-S, Kim Y-S, Shin D-H. 2006. Isolation, Identification, and Fermentation Characteristics of *Bacillus* sp. with High Protease Activity from Traditional Cheonggukjang. *Korean Journal of Food Science and Technology* **38**: 82–87.

20. Greenfield LM, Puissant J, Jones DL. 2021. Synthesis of methods used to assess soil protease activity. *Soil Biol Biochem* **158**: 108277.

21. Rejsek K, Formanek P, Pavelka M. 2008. Estimation of protease activity in soils at low temperatures by casein amendment and with substitution of buffer by demineralized

water. *Amino Acids* **35**: 411–417.

22. Lee R-H, Yang S-J, Hwang T-Y, Chung S-K, Hong J-H. 2015. α-Glucosidase inhibitory activity and protease characteristics produced by *Bacillus amyloliquefaciens*. *Korean Journal of Food Preservation* **22**: 727–734.

23. Lee R-H, Yang S-J, Hong J-H. 2016. Changes in cultural characteristics and biological activities of amaranth during fermentation. *Korean Journal of Food Preservation* **23**: 568–575.

24. Wei C, Su F, Yue H, Song F, Li H. 2023. Spatial distribution characteristics of denitrification functional genes and the environmental drivers in Liaohe estuary wetland. *Environmental Science and Pollution Research*.

25. Tang X, Zou L, Su S, Lu Y, Zhai W, Manzoor M, *et al.* 2021. Long-Term Manure Application Changes Bacterial Communities in Rice Rhizosphere and Arsenic Speciation in Rice Grains. *Environ Sci Technol* **55**: 1555–1565.
